# Weak evidence for neural correlates of task-switching in macaque V1

**DOI:** 10.1152/jn.00085.2022

**Published:** 2023-03-22

**Authors:** Richard D. Lange, Camille Gómez-Laberge, Vladimir K. Berezovskii, Anton Pletenev, Ariana Sherdil, Till Hartmann, Ralf M. Haefner, Richard T. Born

**Affiliations:** ^1^Brain and Cognitive Sciences, Center for Visual Science, University of Rochester, Rochester, New York, United States; ^2^Neurobiology, Harvard Medical School, Boston, Massachusetts, United States

**Keywords:** choice probability, noise correlations, task-switching, V1

## Abstract

A central goal of systems neuroscience is to understand how populations of sensory neurons encode and relay information to the rest of the brain. Three key quantities of interest are *1*) how mean neural activity depends on the stimulus (sensitivity), *2*) how neural activity (co)varies around the mean (noise correlations), and *3*) how predictive these variations are of the subject’s behavior (choice probability). Previous empirical work suggests that both choice probability and noise correlations are affected by task training, with decision-related information fed back to sensory areas and aligned to neural sensitivity on a task-by-task basis. We used Utah arrays to record activity from populations of primary visual cortex (V1) neurons from two macaque monkeys that were trained to switch between two coarse orientation-discrimination tasks. Surprisingly, we find no evidence for significant trial-by-trial changes in noise covariance between tasks, nor do we find a consistent relationship between neural sensitivity and choice probability, despite recording from well-tuned task-sensitive neurons, many of which were histologically confirmed to be in supragranular V1, and despite behavioral evidence that the monkeys switched their strategy between tasks. Thus our data at best provide weak support for the hypothesis that trial-by-trial task-switching induces changes to noise correlations and choice probabilities in V1. However, our data agree with a recent finding of a single “choice axis” across tasks. They also raise the intriguing possibility that choice-related signals in early sensory areas are less indicative of task learning per se and instead reflect perceptual learning that occurs in highly overtrained subjects.

**NEW & NOTEWORTHY** Converging evidence suggests that decision processes affect sensory neural activity, and this has informed numerous theories of neural processing. We set out to replicate and extend previous results on decision-related information and noise correlations in V1 of macaque monkeys. However, in our data, we find little evidence for a number of expected effects. Our null results therefore call attention to differences in task training, stimulus design, recording, and analysis techniques between our and prior studies.

## Graphical abstract

**Figure:**
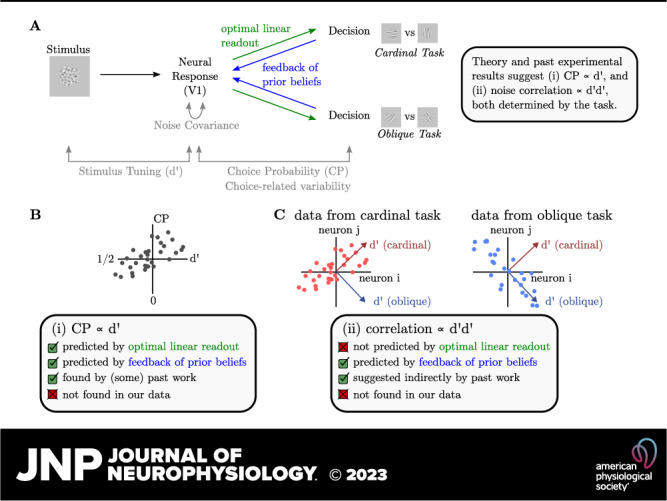

## INTRODUCTION

A central goal of systems neuroscience is to understand how sensory neurons encode and communicate information to the rest of the brain and how information in other areas of the brain, in turn, feeds back to and affects sensory representations. Despite the prevalence of feedback connections among regions of the cerebral cortex, the dominant theoretical framework, based on signal detection theory (1), focuses on feedforward propagation of information.

In perceptual estimation and decision-making contexts, the two most important quantities governing the information in a population of neurons are their sensitivity to the stimulus, quantified by their tuning curves, and correlations in their trial-by-trial variability (2–8). Theoretical work on the importance of so-called “noise correlations” has motivated numerous empirical studies on the nature of correlated variability in sensory cortex (9–11), how it impacts information content (12–15), and how it is impacted by attentional state (16, 17) or other internal states that vary over time (18–20) (see Ref. 21 for a review).

Understanding the neural representations underlying perception requires characterizing both the relationship between stimuli and neural activity as well as the relationship between neural activity and behavior (22, 23). Classically, the latter is quantified using “choice probability” (CP), a measure of how well a subject’s binary decision in a categorization task can be predicted by the fluctuations in activity of single sensory neurons (reviewed in Refs. 23–26). CPs reflect, in part, the causal effect of single neurons on behavior (27, 28), but this interpretation is complicated by the structure of correlated variability between neurons (27–31) and by the presence of cortical feedback containing decision-related information (32, 33).

The interpretation of past results on noise correlations and CPs depends on the extent to which each is due to feedforward or feedback mechanisms. Variable internal states that influence sensory populations induce correlated variability but do not necessarily limit information if the downstream circuits account for them (21, 34). Similarly, partially formed decision states that influence sensory populations induce CPs but do not reflect neurons’ causal roles in the decision (30–33, 35–38). The extent to which noise correlations and CPs are due to feedforward or feedback mechanisms is largely an open question and an active area of research.

Previous studies have found that training on a discrimination task induces a positive correlation between neural sensitivity and CP (39, 40) (reviewed in Refs. 24–26). This relationship between sensitivity and CP is predicted by two distinct theories: optimal feedforward decoding (27, 30, 40) and hierarchical inference (37, 38). Such CP structure has been found across many areas and tasks. In primary visual cortex (V1), this structure has been reported by two previous studies using the same stimulus family as ours (41, 42).

Notably, some other studies did not find a strong positive relationship between neural sensitivity and CP in seemingly comparable experimental designs. Nienborg and Cumming (43) found choice-related information in V2 but not in V1 during a disparity-discrimination task, but in follow-up work the same authors found choice-related information in V1 using a stimulus similar to ours (42). Jasper et al. (44) did not find significant choice information in V1 using high-contrast gratings and a fine-discrimination task. Also using high-contrast gratings and a fine-discrimination task, Goris et al. (45) found mixed results: a negative relationship between CP and neural sensitivity in one animal and no systematic choice-related information in V1 or V2 of another. Using a motion-pulse stimulus and recording from medial temporal cortex, Zhao et al. (46) found that choice-related information was present but uncorrelated with neural sensitivity, lying in a subspace orthogonal to stimulus-related information. Taken together, these studies suggest that the relationship between neural sensitivity and CP is not as straightforward as once believed.

Previous studies on the task-dependent nature of noise correlations have reported significant changes in correlation structure during trial-by-trial task-switching in area MT (47) as well as in V1 with a similar task to ours but without trial-by-trial task-switching (41). Others have found surprisingly high alignment between neural covariability and task sensitivity in single tasks that is difficult to explain by feedforward mechanisms (14, 15, 48). Ours is the first study to investigate the effects on noise correlations in V1 of trial-by-trial cued task-switching.

**Figure 1.**
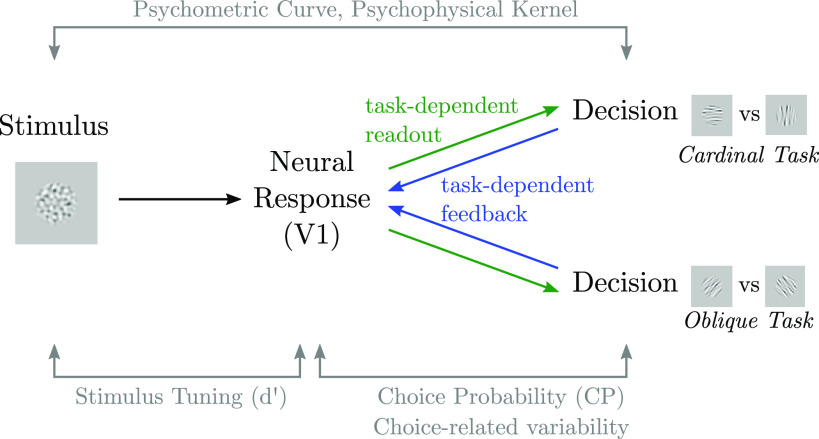
Conceptual overview. We analyze behavior and neural population responses in area V1 of 2 monkeys who switch between 2 randomly interleaved, coarse orientation-discrimination tasks. Switching between tasks, while presenting nearly identical stimuli, isolates task-specific associations between sensory and decision-making areas (37, 41, 47).

We trained two monkeys (*Macaca mulatta*) to perform two coarse orientation-discrimination tasks while we recorded from dozens of units in V1 (Fig. 1). The goal of this experiment was to replicate and extend previous findings on the task-dependent nature of both CPs and noise correlations. Ultimately, we found little choice information in V1 at all, and little evidence for a relationship between neural sensitivity and CP, failing to replicate previous results on patterns of CP. We also found no evidence for changes in noise covariance, failing to replicate previous results on the effects of task-switching on correlated variability. We conclude with a discussion of possible differences between our study and previous ones, including slight experimental differences, idiosyncratic behavior of the monkeys, and differences in analysis methods.

## METHODS

The subjects were two adult male rhesus monkeys (*M. mulatta*) referred to as *monkeys U* (11 kg; 9 yr old) and *A* (10 kg; 8 yr old). All animal procedures were approved by the Institutional Animal Care and Use Committee of Harvard University.

### Orientation-Discrimination Task

Visual stimuli were presented on a 1,600 × 1,200-pixel gamma-corrected cathode ray tube monitor at 85 Hz. Subjects viewed the monitor from 75 cm that subtended visual angles of 29.9° horizontal by 22.6° vertical. The stimulus position away from the fixation point was at (1.90°, −1.80°) for *monkey U* and at (−2.25°, −5.50°) for *monkey A*, with diameters of 2.5° for *monkey U* and 5.0° for *monkey A*. These values were chosen to fully cover the aggregate of all receptive fields. Beyond this diameter, the stimulus faded into the background with soft edges.

The stimulus power spectrum was parametrically controlled with a mask in the Fourier domain. The radial coordinate (i.e., spatial frequency) followed the Rice density with mean of 6 cycles/° for *monkey U* and 4 cycles/° for *monkey A* and with standard deviation of 3 cycles/° for *monkey U* and 1.5 cycles/° for *monkey A*. The angular coordinate (i.e., orientation) followed the von Mises density whose mean parameterized the discriminandum, taking 0° or 90° values for the Cardinal task and 45° or 135° for the Oblique task, and whose concentration parameter κ determined the signal amplitude. Each frame was normalized to have the same maximum deviation between pixel luminance and the mean luminance. Signal levels (*s*) ranged on a scale from –100% to +100% each trial, defined as 1 minus the circular variance of the von Mises density over orientations. A value of *s* = −100% corresponds to a mixture of pure gratings at 0° or 45° with different spatial frequencies, *s* = 0% corresponds to infinite bandwidth with all orientations equally likely, and *s* = +100% corresponds to mixtures of pure gratings at 90° or 135°.

For each trial, the stimulus lasted 1,224 ms with exactly 26 independent white noise images each passed through a fixed band-pass filter (4 frames per image 47 ms). Across trials, we used a frozen-noise strategy for *monkey U* consisting of two sets of three distinct 26-noise-image sequences, the sets alternating between sessions. For *monkey A* in 2017 (*A17*), we used a paired strategy in which each stimulus appeared exactly twice among completed trials. Frozen noise was included to more precisely estimate noise correlations, but this adjustment was found to not significantly impact our results.

A fixed set of 26 signal amplitudes were chosen for both animals ranging from 0% (ambiguous) to 97% (mixture of nearly perfect sinusoidal gratings). Early in training, subjects were exposed exclusively to a subset of the strongest signals (≈90%). As they improved in performance, the subset gradually shifted toward weaker signals. Ambiguous stimuli were presented in at least 10% of trials once the subject performed substantially above chance on the strongest-signal trials.

**Figure 2.**
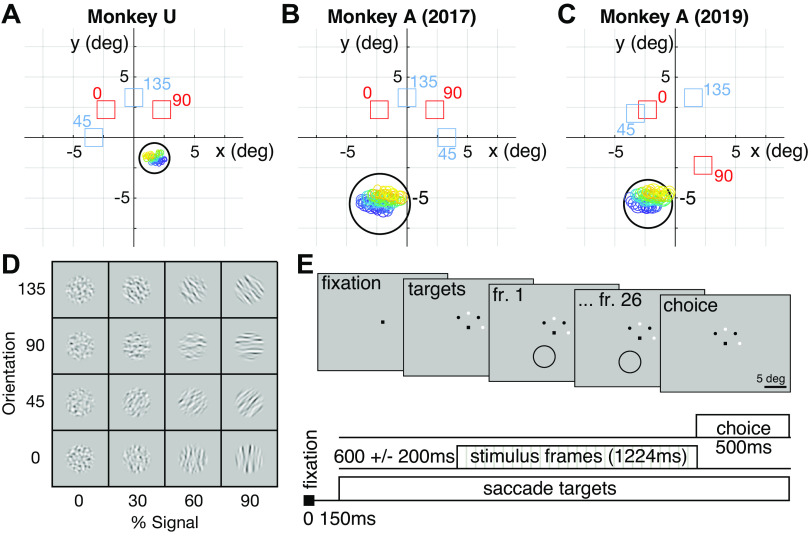
Coarse orientation-discrimination task design and experimental setup. *A–C*: geometry of the display in degrees of visual angle. Fixation point was at the origin. Stimulus size indicated by large black ring, chosen to cover the aggregate receptive fields of all 96 channels of the array (small colored rings; color indicates linear index of position in the array). Red squares show saccade target locations for the Cardinal task, i.e., stimulus orientations 0° and 90°. Blue squares show the same for the Oblique task, i.e., 45° and 135°. None of these annotations appeared on the screen in the experiment (see *E*). Note that *monkey A*’s receptive fields were consistent over the full 2 years. *D*: example band-passed grating stimuli, showing the 4 orientations used in the task at 4 signal levels. *E*: each trial began when the monkey fixated the central point; 150 ms later, either the white or black pair of targets appeared, indicating Oblique or Cardinal task, respectively. After a random delay of 600 ± 200 ms, 26 stimulus frames appeared sequentially, each for 47 ms, at the location indicated by the black circle (which was not visible), lasting a total of 1,224 ms. Once the final frame completed, the monkey had 500 ms to indicate its choice by making a saccade to 1 of the 2 visible targets.

Subjects performed a context-specific orientation-discrimination task. Each trial began with the illumination of a small fixation point (3′ of visual angle) at the center of the screen. The subject was required to fixate throughout the trial until this point was extinguished. After the first 150 ± 50 ms of fixation, two 0.25°-diameter saccade targets appeared in the periphery. Their position and color indicated the task. For *experiments U* and *A17*, black targets due northwest or northeast (north is up) indicated the appearance of either the 0° or 90° signal of the Cardinal task, respectively. White targets due west for *U* (east for *A* to avoid stimulus interference) and due north indicated the appearance of either the 45° or 135° signal of the Oblique task, respectively (Fig. 2, *A*–*C*). The saccade targets remained visible until the end of the trial. The visual stimulus followed the appearance of the saccade targets by 600 ± 200 ms and lasted for 1,224 ms, after which both the stimulus and the fixation point disappeared (the GO cue). The subject then had up to 500 ms to saccade to one of the targets and hold it for 250 ms. Adhering to this behavioral sequence completed the trial. Trials were considered incomplete if a fixation break exceeding 0.75° occurred at any time before GO or if a target was not selected after GO. Fixation breaks triggered the abrupt extinction of all visual stimuli followed by a time-out for the remainder of the trial length. On completed trials, the feedback period lasted ∼500 ms: correct answers were followed by a brief high-pitch tone and water (for *U*; diluted apple juice for *A*) reward, and incorrect trials were followed by a long low-pitch tone. On ambiguous trials (nominal 0% signal), the rewarded response was determined randomly. The intertrial interval was 750 ms, giving a complete trial cycle duration of no more than 4 s. Center of gaze was measured for the right eye with a 1 kHz sampling rate, using an infrared eye tracker (iScan).

We included for behavioral analysis all trials with choice saccades initiated in a window of [–75 ms, 700 ms] relative to stimulus offset and for neural analysis all trials in a window of [+50 ms, 700 ms].

### Inference of Behavioral Strategy

We used the Psignifit library for MATLAB (49) to fit psychometric curves shown in Fig. 4. We set the stationarity prior of the beta-binomial model to 5, where ∞ is a perfectly stationary observer and 1 is a flat prior, and we used a logistic curve over percent signal strength for the link. All other parameters were left at default values. Figure 4 shows the maximum a posteriori (MAP) fits to all completed trials in the −20% to +20% signal strength range, concatenated across sessions per epoch. For fits to all trials, see Supplemental Fig. S2.

We used a form of psychophysical regression to quantify the monkeys’ behavioral strategy in each task. To do this, we first quantified the empirical amount of signal at each orientation in each frame in a trials × orientations × frames array, **S**. Each entry **S***_t_*_θ_*_f_* was computed as the dot product between an orientation-dependent mask or template in the Fourier domain, *M*(θ), and the signal power of the frame at that orientation. Let ρ,ϕ be the spatial frequency and orientation polar coordinates in the Fourier domain; then the orientation-dependent template is given by

$$M{\left(\theta \right)}_{\rho \phi }=\text{Rice}(\rho ;\mu_{\rho },\sigma_{\rho })I_{tf}\left\{\left|\theta -\phi \right|<7.5{}^{\circ}\right\}$$

where Rice() is the density function of a Rician distribution. We used the true spatial frequency statistics, μ_ρ_ and σ_ρ_, from the task. We used 12 templates spaced from θ = 0° to θ = 165° in 15° increments. The signal per trial, orientation, and frame was computed as

Stθf=∑ρϕ‖F(Itf)‖ρϕ2 M(θ)ρϕ

where ${\left\|F(I_{tf})\right\|}_{\rho \phi }^{2}$ indicates the squared norm of the two-dimensional(2-D) Fourier transform of the stimulus that was shown to the monkey, **I***_tf_*, including the soft-edged circular aperture. To standardize regressors, we simulated a large number of zero-signal stimuli to compute the expected mean and variance of **S** at zero signal (which is identical for all orientations θ). We *z*-scored all entries of **S** with respect to the distribution of zero-signal stimuli so that it would be comparable across tasks, orientations, and monkeys. For spatial kernels, we averaged **S** across the frames dimension to get a vector of 12 signal levels each trial, 1 per orientation. Below we refer to this standardized vector of signal levels per trial as **u***_t_*.

The psychophysical kernel model consisted of 15 parameters: 12 weights on the amount of signal at each orientation (**w**_θ_), a bias (**w***_b_*), and two lapse terms (λ_1_ and λ_2_). As in logistic regression, we used a Bernoulli likelihood for each trial, computed as

(*1*) $$z_{t}=w_{b}+\sum_{\theta }w_{\theta }u_{t\theta }$$

(*2*) $$q_{t}=\lambda_{1}+(1-\lambda_{1}-\lambda_{2})\times \sigma (z_{t})$$

(*3*) $${\text{log likelihood}}_{t}=\{\begin{array}{ll} \log q_{t} & \text{if }c_{t}=+1\\ \log(1-q_{t}) & \text{if }c_{t}=-1 \end{array}$$

where σ(*z*) is the sigmoid function, σ(*z*) = [1 + exp(−*z*)]^−1^, *c_t_* is the monkey’s choice on trial *t*, and *q* is the probability of choosing the “positive” orientation.

The prior over spatial weights, **w**_θ_, was implemented as the sum of a ridge penalty of 6 and a second derivative (smoothness) penalty of 0.5. The net effect of these two parameters is equivalent to a Gaussian prior on **w**_θ_ with variance 0.122 per weight and correlation between adjacent entries of **w** beginning at 0.21 and then falling off for larger differences in orientation. The prior on the bias term **w***_b_* was also Gaussian with variance 6.7, and the prior on lapse parameters was exponential with mean 0.06. All of these hyperparameters were chosen using cross-validation, averaged over randomly chosen sizes of the training and validation split, thus maximizing an unbiased estimator of the model evidence (50). This is a stochastic optimization problem for which we used Bayesian adaptive direct search to find the best hyperparameters (51). We performed this evidence maximization procedure separately per session and then took the median of the best-performing hyperparameters (there were no systematic differences across monkeys or epochs).

Inference of the full 15-parameter model was done with MATLAB’s built-in Hamiltonian Monte Carlo (HMC) sampler (52). Sampler hyperparameters were automatically tuned, and samples were manually inspected for convergence. Accounting for slight autocorrelations, HMC typically produced >600 effective samples per parameter for every 1,000 samples drawn. We generated 1,000 samples for most cases but increased this in the rare case where convergence was an issue. Figure 5 shows the mean ±68% posterior intervals estimated from 1,000 samples for the spatial weights and bias. For all kernel-related analyses, we included only trials below 20% signal.

To test for switching strategies during interleaved sessions, shown in Fig. 5, *right*, we randomly split each session into 20 folds for cross-validation. We computed within-task log likelihood (LL) using the best-fit kernel parameters from the 19 “training” folds to predict each held-out “test” fold and then reported the average LL per trial, averaged over training/test splits. We similarly computed across-task LL scores using 19 training folds from one task to predict the held-out test fold from the other task. We are primarily interested in whether the orientation weight parameters, **w**_θ_, switch depending on the cued task, more so than whether the bias or lapse terms change. Furthermore, the sign of **w**_θ_ is arbitrary across tasks, as there is nothing intrinsically relating 90° to 135° more so than 90° to 45°. For these reasons, we constructed a surrogate set of model parameters for across-task predictions by transferring **w**_θ_ between tasks but keeping **w***_b_* and λ from the target task. We used whichever of +**w**_θ_ or −**w**_θ_ performed better. This resulted in 20 “within-task” LL scores and 20 “across-task” LL scores for the same folds. Since we are interested in the difference between within- and across-task scores, Fig. 5, *right*, includes error bars that were computed as the standard error of the mean of LL scores, computed across folds, after projecting the scores onto unit vectors in the *y* = *x* and *y* = −*x* directions.

**Figure 3.**
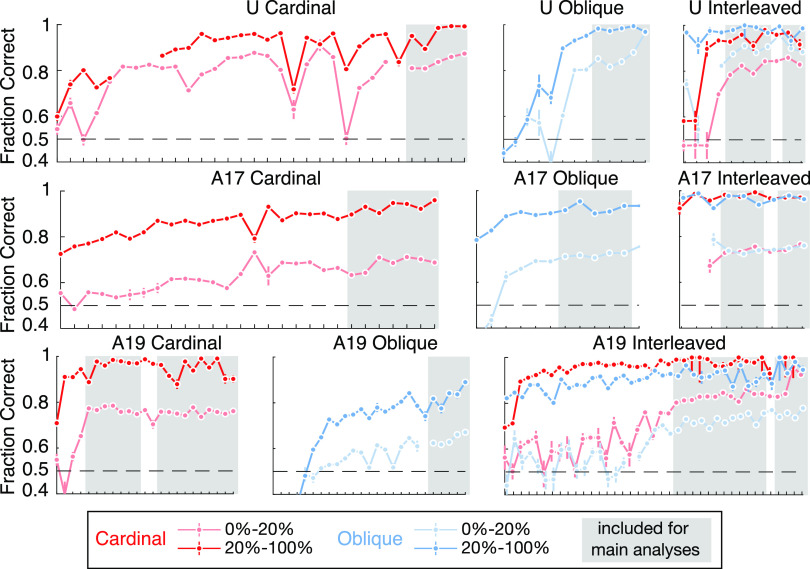
Performance across individual sessions in each epoch, broken down into low (0–20%)- and high (20–100%)-signal trials. Ticks on the *x*-axis are individual sessions. Points and error bars give an estimate of performance in the center of each signal range, i.e. at 10% for the low-signal range and 60% for the high-signal range, respectively. Error bars indicate ±1σ, estimated using the posterior mode and variance from logistic regression. Gray boxes indicate sessions grouped for analysis in the main text. We chose to keep groups of sessions for further analysis based on a combination of performance (as in this figure) and stability of their strategy (Supplemental Fig. S1).

To estimate when behavior had become stable within an epoch, as in Supplemental Fig. S1, we used the full 15-parameter psychophysical kernel model fit to sub-20%-signal trials in each individual session to predict decisions on other sessions in the same epoch. We quantified this cross-session predictability score using the Bernoulli log-likelihood model above, averaged over both trials and samples of model parameters. Because some sessions are intrinsically easier to predict than others (e.g., if they have fewer low-signal trials), we normalized this score by subtracting the log likelihood of the target session predicted with its own psychophysical kernel, resulting in a cross-session behavioral consistency score, plotted in Supplemental Fig. S1 (see also Supplemental Text). We selected which sessions were grouped together per epoch by manual inspection of this cross-session strategy score, psychometric curves, and per-session performance (Fig. 3).

We used simulated choices to verify that the psychophysical kernel models were not simply fitting noise or biased to the particular set of random seeds per group of sessions. To do this, we took the actual distribution of signals, **S**, for each session and passed them through a battery of hypothetical kernels to generate synthetic choices. These artificial kernels included the optimal Cardinal and Oblique discrimination kernels, four “detection” kernels for each orientation, and a mixed-strategy kernel that performs well on both tasks. Across all sessions and hypothetical kernels, we verified that the above fitting routine always recovered a kernel close to the true generating kernel (correlation > 0.9). This ensures that if the monkey had used any of these strategies on any session, we could detect it.

### Electrophysiology

Each subject underwent two operations with aseptic technique and general anesthesia. In the first procedure, a titanium headpost was attached to the skull with 13 self-tapping orthopedic screws. In the second procedure, a 96-channel microelectrode array (MEA) (Blackrock Microsystems) was implanted on the medial region of the occipital operculum in area V1 and three cryoloops (53) were implanted in the lunate sulcus, ipsilateral to the array. In *monkey U*, the implants were placed in the left hemisphere after chair, fixation, and delayed-saccade training. In *monkey A*, the implants were placed in the right hemisphere after chair training only, and an additional 32-channel microelectrode array (Blackrock Microsystems) was implanted 3 mm lateral to the 96-channel array in the same procedure (unused in this study). Analog signals were amplified and sampled at 30 kHz with a 128-channel neural signal processor (Blackrock Microsystems) and stored to a server.

Spikes were processed offline with two passes over the data. In each pass, signals were first high-pass filtered at 300 Hz with a forward and backward pass of a third-order Butterworth filter and then common-average referenced (at each time point, the median signal across channels subtracted). The first pass estimated covariance of the filtered signals across electrodes, whitened them with the ZCA transform, and clipped out threshold crossings at 3σ to remove spikes and other artifacts. In the second pass, we reestimated the covariance of the background fluctuations with threshold crossings from the first pass removed, and signals were again whitened with ZCA. Threshold crossings at 6σ in this second pass were clipped as spikes, with each spike time aligned to the local minimum in the voltage. Artifacts were removed by excluding deflections larger than 20σ relative to background noise and keeping only the smaller of two local minima that appeared within 0.2 ms (6 bins) of each other. Spike waveforms extracted by this procedure were manually inspected in a subset of sessions to ensure that no artifacts remained. No spike sorting was performed, as previous studies similar to ours have reported no significant difference between single-unit and multiunit results (41, 54). One methodological difference with these previous studies is that our “units” comprise all threshold crossings per electrode, whereas their “multiunits” are unsorted threshold crossings that remain after separating out identifiable single units. The two-pass procedure with whitening was inspired by the preprocessing stages in Kilosort (55). Although it is standard practice in high-density recordings, whitening is rarely performed on Utah array data. However, we found that whitening was essential to remove highly correlated background fluctuations across channels that resulted in large fractions of coincident (±0.5 ms) threshold crossings across channels separated by hundreds of micrometers in the brain. In early analyses, these coincident threshold crossings significantly inflated our estimates of spike count correlations (56) but were effectively removed by whitening the raw voltage traces, without significantly impacting firing rates or waveform shapes in most channels.

To select reliable electrodes for further analysis, on each day we first excluded multiunit activity (MUA) with <4 spikes per trial on average (1,050-ms counting window). A large portion of variance in the remaining population was due to global fluctuations that varied slowly, with time constants on the order of minutes, which are known to complicate estimates and interpretation of both noise correlations and choice probabilities (9, 18, 21, 57, 58). To remove slow fluctuations, we fit a nonparametric model to each unit’s responses on each session that described the unit’s activity as a combination of a slow-varying latent factor, smooth tuning to the stimulus strength (Fig. 7, 3rd column), the choice on each trial (to account for choice-related feedback if it is present), and private noise per trial (see *Eq. 4* below). We used this statistical model in two ways: first, we used it for automated outlier detection by removing highly anomalous units and trials from further analysis; second, we used it to subtract an estimate of the slow background fluctuations, effectively isolating independent activity per trial (Fig. 7, 4th column). Among sessions analyzed here, outlier detection flagged and removed between 0 and 36 (median 5) trials with anomalous neural events per session, and it removed between 0 and 80 (median 21) anomalous data points per session from individual units on individual trials. After these preprocessing steps, the median session in *monkey U*’s data had 39 units, *monkey A17*’s had 59, and *monkey A19*’s (*monkey A* in 2019) had 30. We further excluded untuned units, which we identified as those that were not significantly modulated by drifting gratings (*P* > 0.01 by ANOVA), and those whose tuning to drifting gratings, collapsed across drift directions to 180° of orientation, had circular variance > 0.8 (59). This further reduced the min/median/max unit counts per session to 18/27/39 for *monkey U*, 18/31/43 for *monkey A17*, and 14/22/33 for *monkey A19*. For a detailed breakdown of number of units and number of trials per session, see Supplemental Tables S1–S3.

Multiunit spikes in the task were counted in a [150 ms, 1,200 ms] window following stimulus onset. The 150-ms delay accounts for the response time of V1 and onset transients (Fig. 7), and 1,200 ms marks stimulus offset. For all neural analyses, we only included trials in which the monkey’s reaction time was at least 50 ms after stimulus offset to avoid contamination of spike counts by eye movements. To stabilize variance, spike counts were Anscombe transformed (60), defined as

$$r_{i}=2\sqrt{{\text{spike counts}}_{i}+3/8}\;$$

for neuron *i*. The Anscombe transform, like the closely related and more commonly used square-root transform, stabilizes the variance of Poisson random variables to 1 (and super- or sub-Poisson variability to above or below 1, respectively). Because spike counts are typically nearly Poisson distributed, this means that using a Gaussian likelihood model on Anscombe-transformed data is more appropriate than on raw spike counts. We use **r***_t_* to refer to the vector of Anscombe-transformed spike counts on trial *t*.

Initial exploratory analyses revealed that the variance (and covariance) in **r** contained significant slow fluctuations and was inflated by occasional outliers. As briefly mentioned above, we addressed both of these problems by fitting each neuron with a nonparametric model that we refer to as the data-cleanup model. The data-cleanup model describes the response of neuron *i* on trial *t* as the sum of *1*) a baseline rate, *2*) a slowly varying latent factor *x_ti_* times loading factor $w_{i}^{x}$, *3*) tuning to the stimulus *f*(*s_t_*), *4*) influence of the monkey’s choice *c_t_* times a loading factor $w_{i}^{c}$, and *5*) independent noise:

(*4*) $$r_{ti}=b_{i}+x_{ti}w_{i}^{x}+f_{i}^{t}(s_{t})+c_{t}w_{i}^{c}+\epsilon_{ti}$$

We fit separate tuning curves, *f*, for each task. That is, *f^t^*(*s_t_*) = *f^A^*(*s_t_*) when *t* is a cardinal trial and *f^t^*(*s_t_*) = *f^B^*(*s_t_*) when *t* is a oblique trial, so *f^A^*(*s*) and *f^B^*(*s*) are the cardinal and oblique tuning to stimulus strength, respectively. We placed smooth Gaussian process (GP) priors on both *x* and *f* and optimized the parameters of the model using variational Bayes expectation maximization (VBEM). Latent variables include the state of slow fluctuations, *x_t_*, and the tuning curve(s) *f^A^*(*s*) to Cardinal stimuli and *f^B^*(*s*) to Oblique stimuli, where *s_t_* is the scalar percent signal level on trial *t* and ranges from −100% to +100%. The tuning curves were constrained to have the same value at 0% signal, so *f^A^*(0) = *f^B^*(0), and in noninterleaved sessions only a single *f* was used. We imposed a Gaussian process (GP) prior on the slow fluctuations, *x_t_*, using a squared exponential kernel with a timescale of 120 s, reflecting the average learned timescale in initial testing. This kernel was applied to actual wall-time differences between trials to account for varying gaps between trials (e.g., if a trial was removed because of fixation break).

We fit this model separately for each neuron on each day. As in the classic EM algorithm, the VBEM algorithm alternates between the “E-step” and the “M-step” until convergence. The E-step consists of computing

$$\text{q}(x)\text{q}(f^{A})\text{q}(f^{B})\approx \text{p}(x,f^{A},f^{B}|r,s\ldots )$$

where “…” stands for all of the fit parameters of the model. Decomposing the joint distribution over **x** and **f** into a product of marginals is known as the structured mean field (SMF) assumption. We used the mean field variational Bayes (MFVB) algorithm to minimize KL divergence between the SMF factorized posterior and the true posterior conditioned on all observations and parameters. The M-step consists of maximizing a tractable substitute for the data likelihood known as the evidence lower bound (ELBO). The tightness of the bound depends only on how well the SMF factorized posterior approximates the true posterior, and we can expect this to be a reasonable approximation for our model.

Once VBEM fitting converged, we used the model’s predictions to detect outliers in the raw data. First, we excluded units whose Fano factor was >2 after subtracting predicted spike counts from the data-cleanup model (i.e., a Fano Factor > 2 in the residuals not explained by the model), units with mean rates below 4 spikes per trial, and units whose response variance across all trials was <0.1. To calculate the Fano factor, we converted back from **r** to spike counts using the inverse Anscombe transform, which gives the expected mean Poisson rate from the value of **r**, accounting for the bias when taking the expected value of a nonlinear function. The inverse transform is given by

$$E[\text{spike count}]\approx -\frac{1}{8}+\frac{r^{2}}{4}+\frac{1}{4r}\sqrt{\frac{3}{2}}-\frac{11}{8r^{-2}}+\frac{5}{8r^{3}}\sqrt{\frac{3}{2}}$$

From E[spike count] and assuming Poisson spiking, we additionally computed the empirical surprisal of each observed spike count, defined as the log probability of spike counts minus its expected log probability (its entropy). This gives a measure of the surprise of each observation in units of bits. Unlike the probability mass function itself, which becomes more disperse for higher E[spike count], the expected distribution of surprisal values is invariant to the mean of the Poisson. We therefore used a threshold on surprisal bits to detect outliers in three different ways. First, we excluded all trials where at least 10% of remaining units were “surprising” at a threshold of 2 bits. Second, we excluded all units that were “surprising” on at least 10% of trials, also at a threshold of 2 bits. Third, we excluded individual units on individual trials (set their response to “not a number”) if their surprisal exceeded 8 bits.

We repeated the above process in a loop: fit the cleanup model with VBEM, detect and exclude outliers, then refit the model and repeat. When this process converged (or after 10 iterations), we computed final “clean” neural responses, for all units and trials that were not excluded, as

$$r_{i}-{\hat{x}}_{i}w_{i}^{x}$$

where ${\hat{x}}_{i}$ is the MAP (equivalently, the posterior mean) estimate of the latent slow fluctuations for unit *i*. That is, we subtracted our best estimate of slow fluctuations from all Anscombe-transformed responses. These are the data we analyze in the main text.

This data preprocessing pipeline was designed in an early exploratory phase of this work, with the aim of removing confounds and reducing bias. Once this analysis was complete, we reran our main analyses with simpler preprocessing methods and found no qualitative changes to our results. Specifically, we reran all analyses on choice probability, neural sensitivity, and changes to noise covariance structure on neural responses defined as

1)  the firing rate in the [150 ms, 1,200 ms] window, without the Anscombe transform or the Gaussian process model, minus the prestimulus firing rate in a [−500 ms, 0 ms] time window relative to stimulus onset.
2)  the same as the previous but including onset transients by measuring the rate in a [0 ms, 1,200 ms] window and again subtracting the [−500 ms, 0 ms] baseline rate.

As mentioned in discussion, these additional analyses using simpler preprocessing methods resulted in the same or stronger conclusions as in the main text.

Orientation tuning curves to drifting gratings were measured at the start of the day on a subset of sessions. In each trial, three high-contrast drifting gratings were presented (150 ms static, 250 ms drifting, 200 ms blank), with an intertrial interval of 700 ms. All three orientations were randomly selected per group of stimuli per trial, tiling 360° in 15° increments. For all quantities derived from orientation-mapping sessions except for circular variance, spikes were counted in a [50 ms, 350 ms] window and Anscombe transformed to stabilize their variance, as described above. To compute circular variance, we used spike rates in the [50 ms, 350 ms] window and subtracted the baseline rate for each unit, where the baseline was computed from a [0 ms, 30 ms] window after stimulus onset, averaged over all trials (not subtracting the baseline results in inflated circular variance estimates). All main text analyses excluded units whose circular variance during orientation tuning was >0.8, based on the nearest session that included orientation mapping trials (orientation mapping was done every day for *monkey U*; 1 session for *monkey A17* required using the orientation mapping data from the previous day; orientation mapping was done only once for *A19*, so all sessions reused this tuning data). See Supplemental Fig. S5 for a summary of tuning statistics for each monkey.

### Neural Sensitivity and Choice Probability

We defined **f′** as the vector of slopes of each neuron’s tuning curve in the task, measured at *s* = 0. **f′** was calculated using linear regression between **r***_t_* and λ*_f_*tanh(*s_t_*/λ*_f_*). The tanh nonlinearity addresses the fact that neurons were consistently nonlinearly tuned to the percent signal-strength axis (Fig. 7), with firing rates that commonly leveled off around 50% signal in *monkey U* and 80–90% in *monkey A* because of differing eccentricities and spatial frequencies. To estimate tuning slope around 0% signal using only trials above 5.67% signal, it was therefore necessary to rescale the stimulus space such that tuning was approximately linear in the rescaled space. A tanh function worked well and gave nearly identical estimates of tuning slope as the GP fits from the data-cleanup model. The value of λ*_f_* was set to 30 in *monkey U* and 60 in *monkey A*, chosen to minimize residual variance across all sessions. **f′** was estimated using all valid/completed trials above 5.67% signal. This ensured that **f′** was always estimated with a disjoint set of trials from noise covariance, which was estimated with signal levels exclusively below this threshold. To estimate uncertainty in **f′**, we recomputed it 500 times, each time resampling trials with replacement from within this set of trials with absolute value signal >5.67%.

The **d′** vector is defined for neuron *i* as $d\prime_{i}=50f\prime_{i}/\sigma_{i}$, where $f\prime_{i}$ is defined using the tanh method described above and σ*_i_* is the square root of the neuron’s private variance estimated from residual responses per signal level on <5.67% signal trials (i.e., from the diagonal of the noise covariance matrix). Scaling by 50 is done to extrapolate out to ±50% signal, where most tuning curves saturate anyway (see above), so that **d′** estimates the maximum sensitivity of each neuron in the task.

Choice probabilities were computed for each unit by first subtracting the mean response per unique signal level and then combining all residual responses on trials up to ±20% signal. Signal levels with fewer than 20 trials were excluded. We estimated uncertainty in CP values by bootstrap resampling of all trials in this range. For each set of resampled data, we computed CP as the probability that a sample from **r***_i_* among trials when the choice was “positive” was larger than a sample from trials when the choice was “negative.” Significance of individual CP values was estimated from the cumulative distribution function (CDF) of bootstrapped values.

### Mixed-Effects Regression

We used hierarchical mixed-effects regression with measurement error to test for a relationship between CP and **d′** or between each of CP or **d′** across tasks.

Standard least-squares regression makes two assumptions that are inappropriate for our data when analyzing CP and d′ across days. First, standard regression is asymmetric: regressing CP against d′ gives results different from regressing d′ against CP, because an implicit assumption is that the *x* quantity is known precisely and all error is in the *y* quantity. We account for measurement noise in *x* by inferring an underlying latent value of each *x*, and it is this underlying *x* value that drives *y*. Second, standard regression assumes that noise is independent across observations. This is not true in our data because both d′ and, to a lesser extent, CP are correlated across days from the same electrode, or across electrodes on the same day. This is a “crossed” random effects structure that is addressed by linear mixed-effects (LME) models. We combined both of these principles in a single model.

We use the subscript *e* to denote variables that depend on electrode ID only (shared for all sessions), *s* to denote variables that depend on session ID only (shared for all electrodes), and *se* to denote a particular session-electrode combination. Mathematically, we are given pairs of observations (*x*_obs_, *y*_obs_) and estimates of the measurement error, $\sigma_{x,\mathrm{obs}}^{2},\;\sigma_{y,\mathrm{obs}}^{2}$, obtained by bootstrapping the data. We assume that observations are noisy measurements of some latent underlying $\left(\hat{x},\hat{y}\right)$ coordinates:

$$\begin{array}{l} x_{\mathrm{obs}}\;∼\;N(\hat{x},\sigma_{x,\mathrm{obs}}^{2})\\ y_{\mathrm{obs}}\;∼\;N(\hat{y},\sigma_{y,\mathrm{obs}}^{2}) \end{array}$$

where the regression model, $\hat{y}=y_{0}+\hat{\beta }(\hat{x}-x_{0})$, applies to the latent values of $\hat{x}$ and $\hat{y}$. To further account for correlations across electrodes and across days, we introduce additional structure into $\hat{x},\;\hat{y}$, and $\hat{\beta }$. We assume that each $\hat{x}$ value reflects the combination of *1*) an underlying value per electrode *x_e_* drawn from $N(x_{0},\sigma_{x,e}^{2})$, *2*) a session-by-session Δ*x_s_* drawn from $N(0,\sigma_{x,s}^{2})$, and *3*) private noise Δ*x_se_* drawn from $N(0,\sigma_{x,se}^{2})$. In total, each point’s latent x-coordinate is defined by

$$\hat{x}=x_{e}+\Delta x_{s}+\Delta x_{se}$$

Similarly, for $\hat{y}$, we assume

$$\hat{y}=y_{0}+\Delta y_{e}+\Delta y_{s}+\Delta y_{se}+\hat{\beta }(\hat{x}-x_{0})$$

In other words, the net variations on $\hat{x}$ act on $\hat{y}$ through $\hat{\beta }$, but there is further coordinated variation in $\hat{y}$ among data points from the same electrode or same session. Finally, we assume that there may be some day-by-day or electrode-by-electrode variation in the regression slope itself, and so

$$\hat{\beta }=\beta +\Delta \beta_{e}+\Delta \beta_{s}$$

As is standard practice in LME regression, each Δ term was itself assumed to be distributed according to a zero-mean Gaussian whose standard deviation was itself drawn from a half-Cauchy distribution:

$$\begin{array}{l} \Delta x_{s}\;∼\;N(0,\sigma_{x,s}^{2})\\ \sigma_{x,s}\;∼\;\text{HalfCauchy}(0,\sigma_{\sigma xs}) \end{array}$$

and similarly for *y* and β in place of *x* and *e* and *se* in place of *s*. The HalfCauchy prior encourages each σ term to be small, which then encourages the model to explain as little variance as possible with each of these Δ terms, pushing many toward zero and only keeping those random effects that are sufficiently supported by the data. Following common practice, we set the width of each HalfCauchy before five times the standard deviation of the data along the corresponding axis. For the prior over random slopes, σ_β,_*_s_* and σ_β,_*_e_*, we set the width of the HalfCauchy before the ratio of *y* to *x* standard deviations in the raw data.

We used a No U-Turns Sampler implemented in Stan via MatlabStan (61) to infer a joint posterior distribution over all latent variables and model parameters, including β, $\hat{x},\;\hat{y}$, all Δ parameters, and their standard deviations. Source code for the Stan model we used is available in the Supplemental Text.

Whereas the prior for each Δβ*_e_* and Δβ*_s_* has mean 0, the posterior for each may deviate. In an extreme but hypothetical scenario, all β*_e_* could be inferred to be positive, compensated by the opposite bias in the fixed effect β. Both for visualization purposes and for statistical tests, we therefore took the average random effect and added it to the fixed effect, separately per sample. For example, the effective slope parameter from sample *i* was calculated as

$$\beta_{\mathrm{eff}}^{i}=\beta^{i}+\frac{1}{n_{s}}\sum_{s}\Delta \beta_{s}^{i}+\frac{1}{n_{e}}\sum_{e}\Delta \beta_{e}^{i}$$

where *n*_s_ is the number of sessions and *n*_e_ is the number of electrodes. We computed “probability of direction” (p.d.) values for all regressions as the fraction of samples of the slope parameter, β_eff_, combined across fixed and average random effects as just described, that were on either side of zero. For CP versus d′ analyses (Fig. 8), we used a one-sided test, i.e., reporting the fraction of posterior samples where $\beta_{\mathrm{eff}}^{i}<0$. For CP vs. CP and d′ vs. d′ (Fig. 9), we used two-sided tests, i.e., reporting either the of posterior samples where $\beta_{\mathrm{eff}}^{i}<0$ or where $\beta_{\mathrm{eff}}^{i}>0$, whichever was smaller. (Note that we are breaking with convention slightly by reporting probability of direction for the opposite direction of the hypothesis we are interested in so that values are numerically comparable to *P* values without further conversion).

Calculating the fraction of variance explained or *R*^2^ in mixed-effects regression models is nontrivial (63, 65) and is complicated by the fact that we compute a full Bayesian posterior over parameters (62) and the fact that we account for errors in both the independent and dependent variables. We calculated an approximate value for *R*^2^ by combining a few existing techniques and report their values in Figs. 8 and 9. Specifically, we computed an *R*^2^ value separately for each posterior sample and report the median across all samples. For each value of *R*^2^, we used the “marginal” method of (63, 65), defined as

(*5*) $$R^{2}=\frac{\sigma_{\text{fixed}}^{2}}{\sigma_{\text{fixed}}^{2}+\sigma_{\text{random}}^{2}+\sigma_{\text{noise}}^{2}}$$

Specifically, in our model we used

(*6*) $$\sigma_{\text{fixed}}^{2}=\text{var}\left[\beta_{\mathrm{eff}}\left(\hat{x}-x_{0}\right)\right]$$

(*7*) $$\begin{array}{l} \sigma_{\text{random}}^{2}=\sigma_{y,e}^{2}+\sigma_{y,s}^{2}\\ +\frac{1}{N}\sum_{i=1}^{N}{\left({\hat{x}}_{i}-x_{0}\right)}^{2}(\sigma_{\beta ,s}^{2}+\sigma_{\beta ,e}^{2}) \end{array}$$

(*8*) $$\sigma_{\text{noise}}^{2}=\sigma_{y,se}^{2}+\frac{1}{N}\sum_{i=1}^{N}\sigma_{y_{i},\mathrm{obs}}^{2}$$

where $\text{var}(z)=\frac{1}{N-1}\sum_{i=1}^{N}\left(z_{i}-\bar{z}\right)$ is the sample variance. *Equation 6* is the sample variance in *y* explained by the fixed effect, i.e., variation in *x* multiplied by β_eff_. *Equation 7* is the model’s estimate of the total variance in *y* due to random intercept effects (first line) and the average of random slope effects (bottom line) (63). *Equation 7* is the model’s estimate of unexplained variance or noise, given by the sum of average observation noise and the residual “private” noise magnitude. Note that this calculation of *R*^2^ only accounts for variance explained in *y*, not in *x*.

### Noise Correlations and Limited Range Structure

We estimated all values in the limited range correlations analysis by bootstrapping. Preferred orientations were computed from orientation tuning sessions by computing a weighted circular mean, where stimulus motion directions were weighted by neural responses. We combined data across phase directions to reduce 360° motion directions to 180° of orientations and then multiplied all orientations by 2 to compute the circular mean (59), before finally dividing by 2 to again to recover the mean in a 180°-periodic space. We repeated this 500 times while resampling trials from the orientation mapping sessions with replacement. In *monkeys U* and *A17*’s data analyzed here, orientation tuning was measured frequently, no more than 2 days apart from any data analyzed from the task.

For noise correlations, we first subtracted the mean response for each neuron from all trials per unique signal level, excluding signal levels with <5 trials. We then resampled all trials below 5.67% signal with replacement and computed the sample correlation of neural responses for each.

For each pair of units on each session, we had 500 bootstrapped estimates of their difference in preferred tuning, whether they were part of the same pool, and their noise correlation. We concatenated these data across sessions. Mean correlation plots (Fig. 11, *A* and *B*) were computed by binning differences in preferred orientation in 5° bins and then computing the mean and standard error of all correlation values across all bootstraps in that bin; the standard error was computed as the standard deviation of the mean correlation per bin across bootstrap samples, but we did not additionally adjust for repeated pairs of electrodes across days.

Whether a pair was in the “same pool” or “different pool” was determined by whether the sign of their tuning slopes agreed: where ***f′****_i_****f′****_j_* > 0, units *i* and *j* were placed into the same pool. For the analysis designed for comparison with the results of Cohen and Newsome (47), we included only pairs that were in the same pool in one task and different pools in the other task. This ensured that both pools had a matched distribution of units, since each pair appeared in each pool exactly once.

### Changes in Noise Covariance

When there are few trials relative to the size of the neural population, estimating the noise covariance matrix **C** requires regularization. We used the “diagonal” regularizer of Yatsenko et al. (66), defined as $C=\hat{R}⊙\hat{\sigma }{\hat{\sigma }}^{⊤}$ where $\hat{R}$ is a regularized version of the sample correlation matrix (**R**), $\hat{\sigma }$ is a regularized standard deviation per neuron, and ⊙ denotes element-wise matrix multiplication. Regularization is achieved by

$$\begin{array}{l} \hat{R}=R(1-\lambda_{R})+I\lambda_{R}\\ \hat{\sigma_{i}^{2}}=\sigma_{i}^{2}(1-\lambda_{\sigma })+\text{median}(\sigma )\lambda_{\sigma } \end{array}$$

In other words, the private variances of each neuron are shrunk by λ_σ_ toward the average variance and the sample correlations are shrunk by λ*_R_* toward the identity matrix. Through cross-validation, we determined that λ*_R_* = 0.6 and λ_σ_ = 0.1 generalized best to unseen data across sessions and monkeys using the log-likelihood metric described in the following paragraph.

For our model-free metric of changes in covariance, we split neural responses from all low-signal (≤5.67% signal) trials into 100 folds, separately per task. For each fold, we used the training 99% of trials to estimate **C**_train_, using the regularization just described. On the 1% of held-out trials, we estimated per-unit predictability using the multivariate Gaussian log likelihood. Using the sample covariance of held out trials, $C_{\text{test}}^{\text{sample}}$, which can be computed as

log L(rtest|Ctrain)=−12N[Tr(Ctrain−1Ctestsample)+ log|Ctrain|)]

where *N* is the number of units in the population (66). This gave 100 “within-task” log likelihood values per session, separately for each task. We used the same 100 folds to compute the average log likelihood “across tasks,” given by the difference in log likelihood score on the same set of held-out spikes, computed using **C**_train_ from the two different tasks. These are the values plotted in Fig. 12, *B*–*D*. Because these two measures are paired (i.e., reusing the same folds for the within- and across-task scores), we computed 100 “switching scores” for each task and each session, defined as the difference between within- and across-task log likelihood values on each fold. We used a one-sided *t* test to test whether the distribution of switching scores was significantly above zero for each task and each session; no test reached significance.

For the variance analysis in Fig. 12, *E*–*H*, we computed both **f′** in each task as well as the noise covariance 500 times while resampling trials with replacement. As in our other analyses, **f′** and **C** were estimated using disjoint sets of trials with methods described above. For each bootstrapped sample, we normalized **f′** to a unit vector and computed the variance along it as $f\prime^{\text{⊤}}Cf\prime /{\left\|f\prime \right\|}^{2}$. We then repeated this using the **f′** vector from the other task. This resulted in 4 × 500 measurements per session: 500 bootstrap repeats of variance in task *i* along the axis defined by **f′** in task *j*, for all four combinations of *i* and *j* from Cardinal and Oblique tasks. We took differences per axis, i.e., subtracting $f\prime_{i}^{⊤}C_{j}f\prime_{i}/{\left\|f\prime_{i}\right\|}^{2}$ from $f\prime_{i}^{⊤}C_{i}f\prime_{i}/{\left\|f\prime_{i}\right\|}^{2}$, and computed significance of differences per session by the fraction of bootstrap samples below (or above) zero. We concatenated bootstrap samples across sessions to compute significance of variance differences per task and per monkey, none of which reached significance.

### Histology

When all experiments had been completed, we euthanized both monkeys with an overdose of Euthasol (130 mg/kg; Patterson Veterinary), followed by perfusion through the heart with 1 L of rinse solution (0.9% sodium chloride + 0.5% sodium nitrite) followed by 3 L of fixative [4% paraformaldehyde in 0.1 M phosphate buffer (PB), pH 7.4]. For postfixation steps, we left the brain within the skull; however, we removed a large section of the ventral surface of the skull to facilitate the access of solutions to the brain. The brain/skull was postfixed in the same fixative for 24–48 h at 4°C and then immersed in 15% sucrose in 0.1 M PB for 2 days, followed by 30% sucrose for another 5–7 days. After cutting the cable that linked the MEA to the pedestal connector, we carefully removed the pedestal and other surface hardware along with the skull except for the part located immediately above the MEA. After removing the brain from the skull, we placed it in 30% sucrose solution for another 2–3 days at 4°C. Gentle separation of the remaining piece of skull from the underlying dura was done with a spatula, leaving the MEA in place. We then trimmed the dura around the MEA, using extreme caution so as not to disturb the MEA, which remained firmly attached to the cortex (Supplemental Fig. S4a). Next, we centered the entire hemisphere, with the MEA still in place, on the stage of a freezing microtome (medial surface down, to make parasagittal sections) and froze it to −25°C (Supplemental Fig. S4b). Once the tissue block was completely frozen, we removed the MEA by first gently warming the exposed surface of the MEA, using a plastic pipette bulb filled with warm water, then used a forceps to pull it out with a firm, smooth motion (Supplemental Fig. S4c), filling the exposed location of the MEA with OCT (Optimal Cut Temperature, standard medium to embed tissue for cutting). We then cut serial 50-μm parasagittal sections and collected them in 0.1 M PB, processing alternate sections for cytochrome oxidase (67) or Nissl staining using a 0.5% aqueous solution of thionin (Fisher Scientific). Before using this procedure on the two monkeys from our behavioral experiments (*monkeys U* and *A*), we tested it on a different monkey (*monkey V*) that also had a chronic Utah array implanted for >4 yr. For this monkey, 24 h before euthanasia and perfusion, we made electrolytic lesions on two of the electrodes (e9 and e88) by passing direct current (5 μA for 8 s, electrode negative). This produced characteristic lesions that were clearly visible on histological sections (Supplemental Fig. S4d). The characteristic pattern of conical defects at the location of each electrode suggests that the lesions were unnecessary. Nevertheless, it was reassuring to see that the two lesions corresponded precisely to the positions of the two electrodes mapped by the pattern of electrode defects and that the laminar location of the lesions, made while the arrays were still located in the living brain, corresponded to the tips of the defects remaining in the histological sections (Supplemental Fig. S4d).

### Data, Code, and Supplemental Materials

Data, code, and supplemental text, figures, and tables are available publicly online at https://osf.io/nfta6/.

## RESULTS

Results are structured as follows. First, we describe the task, the experimental protocol, and the monkeys’ behavioral performance and task strategy at the end of each training epoch. Next, we turn to neural data, showing first that the recorded neurons are consistent with expected characteristics of V1 neurons including tuning curves, response timing, and the distribution of noise correlations. Finally, we turn to CPs and noise correlations, finding little evidence for task-dependent structure in either.

### Task Training and Behavior

We trained two male Rhesus macaques (*M. mulatta*), referred to as *monkey U* and *monkey A* throughout, to perform two coarse orientation-discrimination tasks: Cardinal and Oblique. The geometry of the screen and the location of the stimulus relative to neurons’ receptive fields are shown in Fig. 2, *A*–*C*. We used “band-passed grating” stimuli, consisting of a random mixture of gratings from a range of spatial frequencies, phases, and orientations (68, 69). In the Cardinal task, the monkeys discriminated between random sequences of vertical and horizontal stimuli, and in the Oblique task they discriminated between random sequences of 45°- and 135°-oriented stimuli (Fig. 2*D*). Task difficulty was controlled by varying the orientation coherence, or bandwidth of orientations mixed into each frame. Coherence was fixed for each trial. We use the arbitrary convention throughout that signal strength ranges from −100% to +100% in each task, with negative signal corresponding to 0° or 45° and positive signal corresponding to 90° or 135° depending on the task context. The stimulus bandwidth parameter is inversely related to percent signal: high signal is low bandwidth and vice versa. An important feature of this stimulus is its that at infinite bandwidth (0% signal), images contain a high-contrast mix of all orientations but a narrow band of spatial frequencies, so that zero-signal stimuli are identical in both Cardinal and Oblique tasks but still evoke strong responses from V1 neurons. Previous work suggests that this stimulus can evoke both choice probabilities (41, 42) and structured noise correlations (41) in V1.

Each trial began with the monkey fixating a target at the center of the screen. After a short delay, two choice targets appeared whose color and location indicated the discriminanda for that trial (Fig. 2*E*). After a delay of 600 ± 200 ms, the stimulus appeared at a peripheral location matched to the receptive fields of the recorded neurons (Fig. 2, *A*–*C*), consisting of 26 frames that lasted for a fixed duration of 1,224 ms. The stimulus for *monkey A* was more peripheral than that for *monkey U*, and its size and spatial frequency were adjusted accordingly to match the preferences of those neurons. Once the stimulus disappeared, the monkey had 500 ms to indicate its choice with a saccade to one of the two choice targets.

Both monkeys followed a similar training curriculum, which was split into four epochs, where each epoch consisted of multiple sessions. In the first epoch the monkeys were trained exclusively on the Cardinal task, and in the second they were trained exclusively on the Oblique task. Epochs ended when the monkeys’ performance was consistently high for multiple sessions. In the third epoch, Cardinal and Oblique trials were interleaved, initially in blocks and then randomly interleaved. The monkeys were cued to the trial type by the color and location of the saccade targets. In a fourth and final epoch, the monkeys continued in the interleaved paradigm while V2 was reversibly inactivated by cooling to temporarily suppress feedback to V1 (53); however, cooling data are excluded from this paper to manage scope. *Monkey A* underwent the complete experimental paradigm twice, once in 2017 and once in 2019, and his performance had reverted to baseline at the start of retraining in 2019. We report these experiments separately as *A17* and *A19*. Figure 3 shows the training curriculum and performance over sessions.

Neural and behavioral data were recorded throughout training, but we restrict this paper to sessions with stable behavior at the end of each training epoch. We selected groups of sessions based on two criteria: first, we kept only sessions where performance on difficult trials was high (Fig. 3) and second, we kept only sessions with stable behavioral strategy (Supplemental Fig. S1). We thus report three epochs for *monkey U* (Cardinal: 6 sessions, Oblique: 7 sessions, and Interleaved: 7 sessions) and six for *monkey A* (*A17* Cardinal: 5 sessions, *A17* Oblique: 8 sessions, *A17* Interleaved: 6 sessions, *A19* Cardinal: 16 sessions, *A19* Oblique: 7 sessions, and *A19* Interleaved: 16 sessions). Supplemental Tables S1–S3 give further details on the number of trials per session, broken down by signal level.

**Figure 4.**
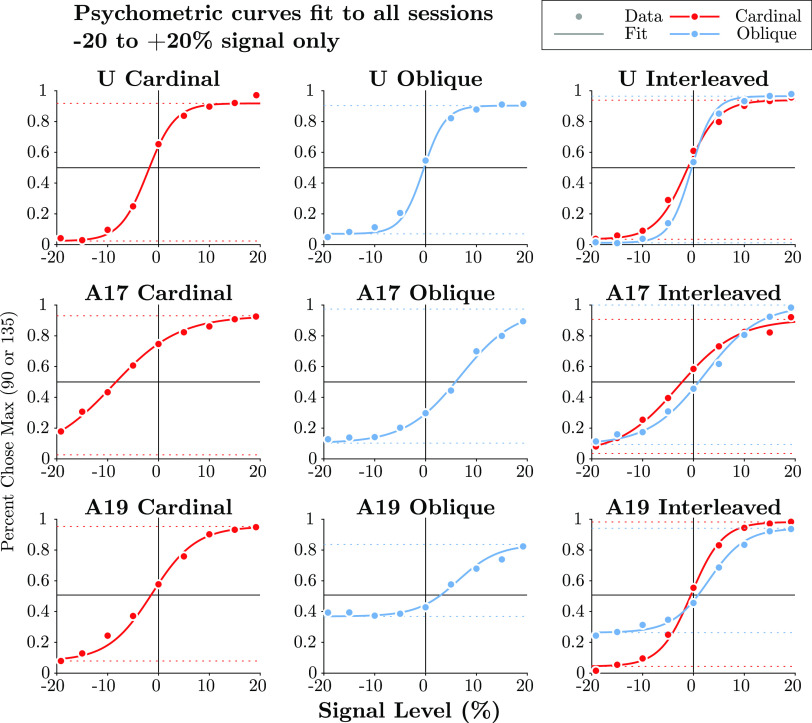
Psychometric curves for sessions at the end of each epoch, zoomed in and fit only to the −20% to +20% signal range. The “Max” choice refers to 90° in the Cardinal task and 135° in the Oblique task, with task types indicated by color. Circles show mean choice pooled across sessions and binned at 5% signal increments. 95% Confidence intervals for the binomial distribution are smaller than the marker size for all points. Curves and dotted lines show the best fit psychometric curve and lapse rates found with the Psignifit toolbox (Ref. 49; methods). Psychometric curves out to ±100% signal are shown in Supplemental Fig. S2.

The monkeys’ average performance at the end of each epoch is shown in Fig. 4. Both monkeys reached stable decision strategies that consistently earned rewards in the task but showed idiosyncratic biases. Overall, *monkey U* was more sensitive and less biased and had lower lapse rates than *monkey A*; the difference in sensitivity is easily explained by the more peripheral stimulus seen by *monkey A* (Fig. 2). In its 2019 retraining, *monkey A* showed a significant asymmetric lapse in Oblique trials, rarely selecting the 45° option, and this bias persisted despite additional focused training trials. One possibility is that this asymmetric lapse reflected confusion about the choice targets: the location of the 0° target on Cardinal trials was close to the location of the 45° target on Oblique trials, raising the possibility that *monkey A* avoided the 45° target because of confusion with the 0° target (but note that they differed in color depending on the task).

### Psychophysical Reverse Correlation Reveals Diverse Decision Strategies

We next quantified whether the monkeys were actually performing the intended discrimination tasks or if they were using a similar but suboptimal strategy. In principle, diverse strategies are consistent with good performance on the task. For instance, in the Cardinal task, the monkey could explicitly look for a vertical grating and then report horizontal only in the absence of evidence for vertical; this would be a “detection” rather than “discrimination” strategy. The signature of this strategy in the data would be an equal likelihood of horizontal choices on trials with matched vertical but differing horizontal evidence. Similarly, a monkey could achieve good performance during interleaved epochs without actually “task-switching,” for instance by grouping 0° and 45°, as well as 90° and 135°, into a single category internally and then applying the same decision rule on all trials.

To determine their strategy, we regressed the monkeys’ choices against the total signal per trial in each of 12 orientation bins spaced uniformly from 0° to 165° in 15° increments. The regression weights across orientations are known as the monkey’s psychophysical kernel. This psychophysical kernel model consists of 15 parameters in total: one for each orientation, a bias term, and asymmetric lapse terms. We used Hamiltonian Monte Carlo sampling to compute the joint posterior distribution over all parameters of the model (52), using a smoothness prior that encouraged weights at adjacent orientations to be similar. We verified in simulations that this method could recover ground-truth kernels from any strategy (methods).

**Figure 5.**
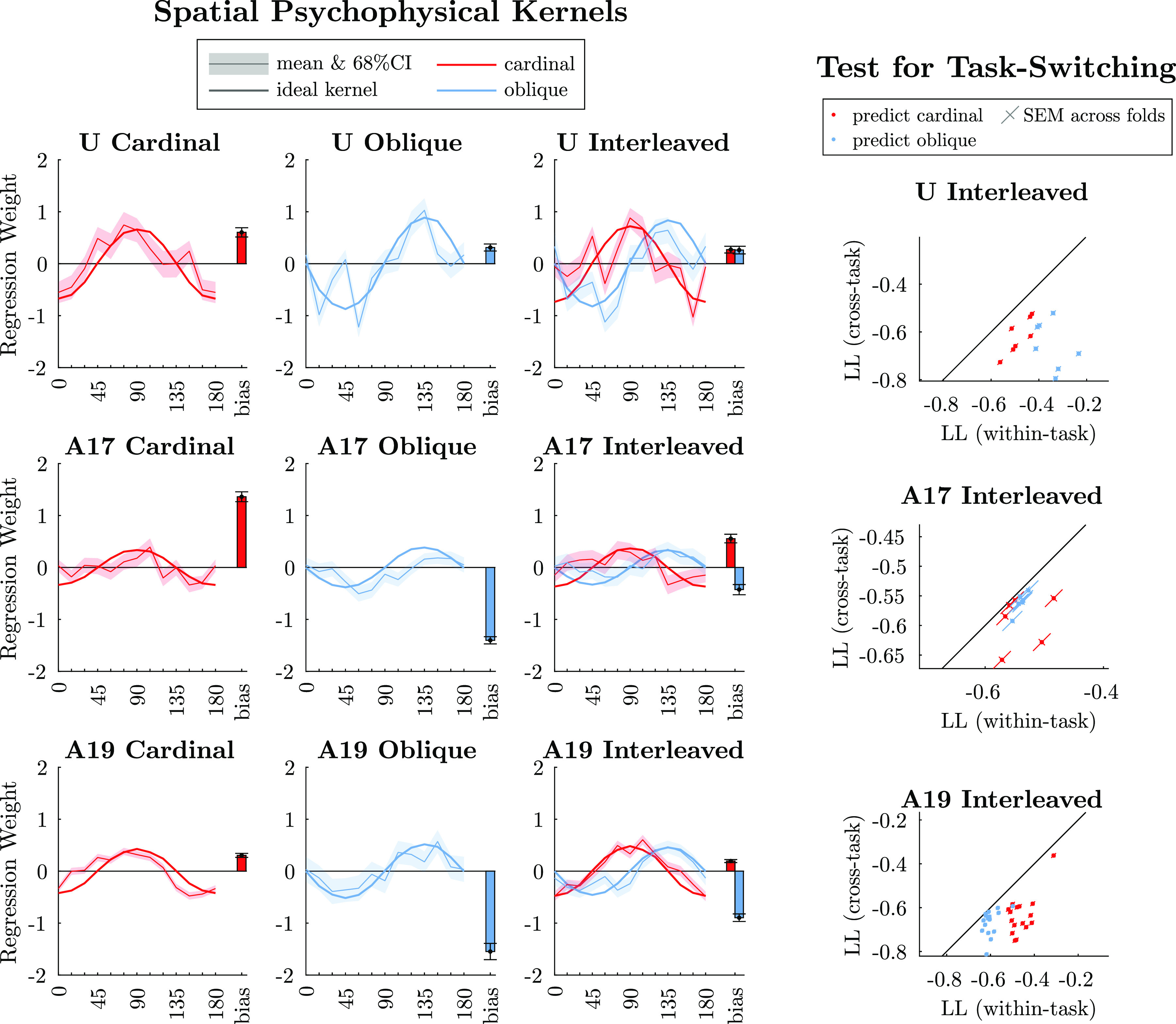
*Left*: task performance is supported by various strategies that resemble but deviate from the ideal observer, with a variety of biases. We fit a 15-parameter regularized logistic regression model to subject’s choices, including 12 weights on oriented stimulus energy equally spaced from 0° to 165°, an upper and lower lapse rate, and a bias term (methods). Each panel plots weights against orientation energy in the stimulus [faint line and shading are posterior mean and 68% credible intervals (CIs), i.e. the central 68% of the posterior mass]. Magnitude of the weights is relative to *z*-scored signals (methods). The ideal observer’s discrimination weights are plotted as thick lines for comparison. Colors are as in previous figures (red is Cardinal task, blue is Oblique task). Bars show the model’s bias toward the positive choice (i.e. regression weight against a constant set to 1). *Right*: for each interleaved session, we tested the ability of the psychophysical kernel in one task to predict choices in the other task, using cross-validation with 95% training and 5% test trials. The more the monkey’s strategy switches between tasks, the greater log likelihood (LL) should be within rather than across tasks. We see greater evidence for switching by *monkeys U* and *A19* and evidence of a constant strategy by *A17*.

Results of this analysis are shown in Fig. 5, *left*. All inferred kernels are consistent with the monkeys’ psychometric curves (Supplemental Fig. S3). Overall, it is clear that the monkeys’ strategies resemble but subtly differ from the optimal discrimination kernels. Two systematic deviations are worth noting. First, for *A19* Cardinal, it appears that the monkey is internally discriminating targets that are rotated 15° to 30° counterclockwise from the true Cardinal targets. Second, the two kernels for *A17* Interleaved are nearly mirror images of each other: both are consistent with detecting 90° or 135° as a single pattern while largely ignoring 0° and 45° signals. Because the sign of the kernel is based on the arbitrary convention of treating 0° and 45° as “negative” and 90° and 135° as “positive,” this mirror-image structure in *A17* Interleaved indicates that the monkey may have applied the same or similar strategy in both tasks.

To quantify the extent to which the monkeys’ strategies actually switched depending on the task cue during interleaved sessions, we tested whether the spatial kernel inferred for one task would transfer to the other task, optionally flipping its sign, without transferring the lapse and bias parameters. For each session, we compared the log likelihood (LL) of model predictions on held-out trials within the same task type versus the LL of predictions for trials in the other task type. Figure 5, *right*, shows the across-task LL versus within-task LL for each interleaved session. The fact that within-task predictions are larger than across-task predictions in *monkey U*’s and *monkey A19*’s data indicates that *monkeys U* and *A19* employed a different strategy depending on the cued task type. *Monkey A17*’s choices, on the other hand, were nearly as predictable using the kernel inferred for the other task.

These behavioral analyses indicate that although both monkeys learned to perform both tasks well during interleaved epochs, their strategies were not always consistent with each other, or even within the same monkey after relearning the task. However, *monkeys U* and *A19* both switched their strategy between the cued tasks, suggesting that we might reasonably expect task-dependent changes in neural responses.

### Neural Recordings and Anatomy

**Figure 6.**
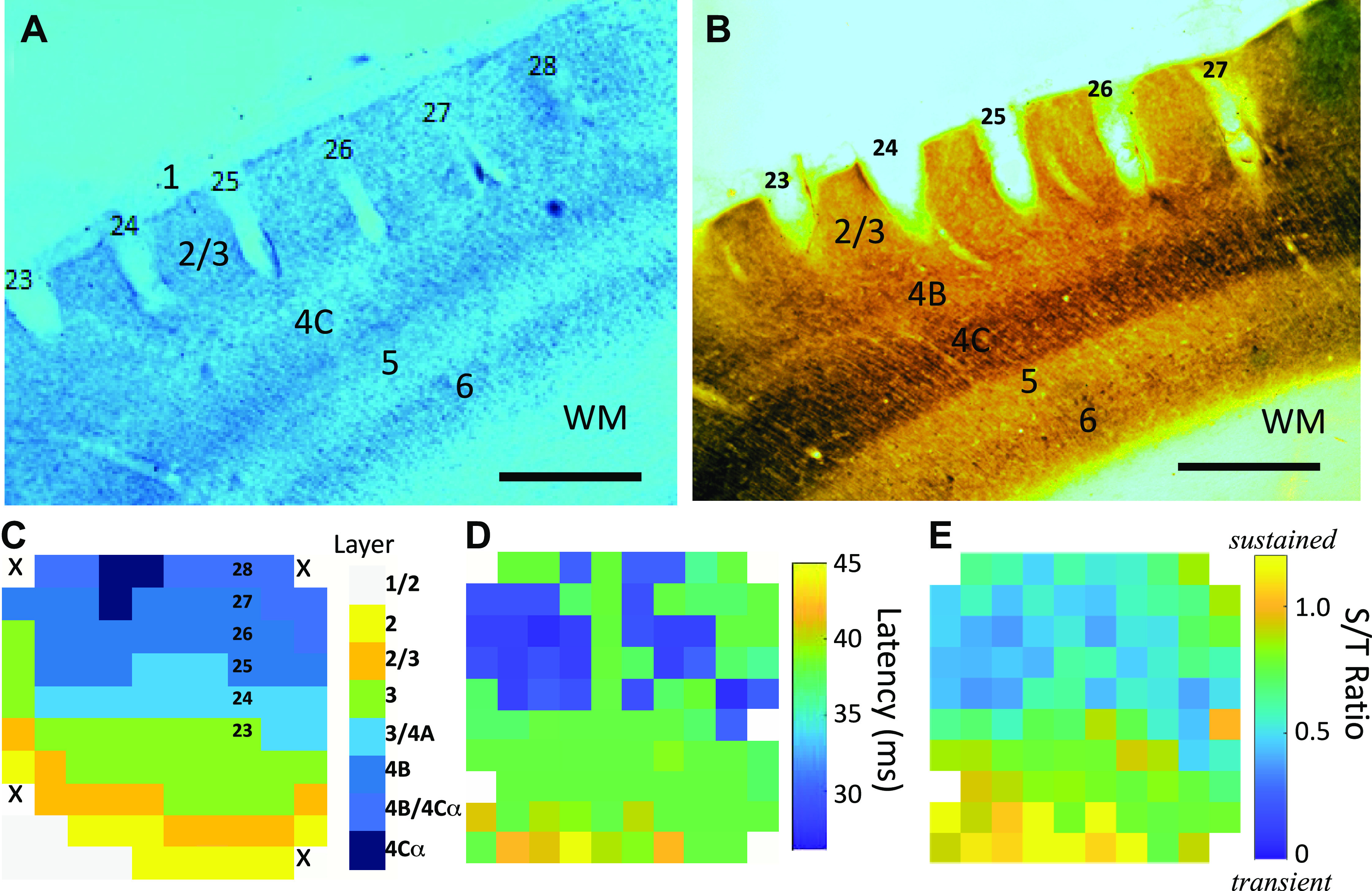
Histology; see Supplemental Fig. S4 for additional details. WM, white matter. *A*: *monkey U*, *section 127* (Nissl). Scale bar, 500 μm. *B*: *monkey U*, section 126 (cytochrome oxidase). Scale bar, 500 μm. *C*: *monkey U*, estimated layer assignments on the array based on histology. *D*: *monkey U*, latency map on the array. *E*: *monkey U*, sustained (S)-to-transient (T) ratio map on the array.

We recorded from Utah arrays (Blackrock Microsystems) chronically implanted in V1, which consist of 96 extracellular electrodes spaced 400 μm apart. Using a combination of Nissl staining (Fig. 6*A*) and cytochrome oxidase histochemistry (Fig. 6*B*), one of us (V.K.B.), who was blind to the results from behavioral and physiology experiments, assigned each electrode to a cortical layer or, if ambiguous, the border between two layers (Fig. 6*C*). From previous analysis of a third monkey (*monkey V*) that did not participate in this experiment, we had made electrolytic lesions on two of the electrodes, which made clear that the deepest discernable tip of the conical defect left by the electrode corresponded to the center of the lesion (Supplemental Figure S4d). We found this point for each electrode and used it to assign that electrode to a layer. Our laminar assignments were in good general agreement with known physiological properties, such as latency of response to visual stimuli (Fig. 6*D*) and the ratio of the magnitude of the “transient” response (the first 40 ms of the neuronal response to a visual stimulus) to the “sustained” component (100 to 1,800 ms; Fig. 6*E*). Although the correspondence was not perfect, as might be expected for recordings that were made several months before euthanasia and histological processing, it was clear that for *monkey U* the upper part of the array was mainly recording from layers 4B and 4Cα, where the visual responses had shorter latencies (Fig. 6*D*) and were more transient, as would be expected from layers that are dominated by magnocellular inputs (70). Conversely, the response properties on the lower half of the array, with longer latencies and more sustained responses, were consistent with the assignment to layers 2/3, which are more influenced by the parvocellular stream. We were somewhat surprised by the clear evidence that we had recorded from layer 4B and below: Given that the length of the electrodes on the arrays was 1 mm, we had anticipated that all of our recordings would be confined to layers 2 and 3. It appears, however, that a combination of *1*) the unintentional insertion of the MEA at a slight angle to the surface of the cortex (Fig. 6, *A* and *B*) and *2*) the possible partial compression of the cortex by the array over time led to these deeper recordings. In *monkey A* (data not shown), both histology and physiological properties were consistent with virtually all electrodes being within layers 2/3, more in line with our prior expectations.

### Preprocessing of Neural Data

**Figure 7.**
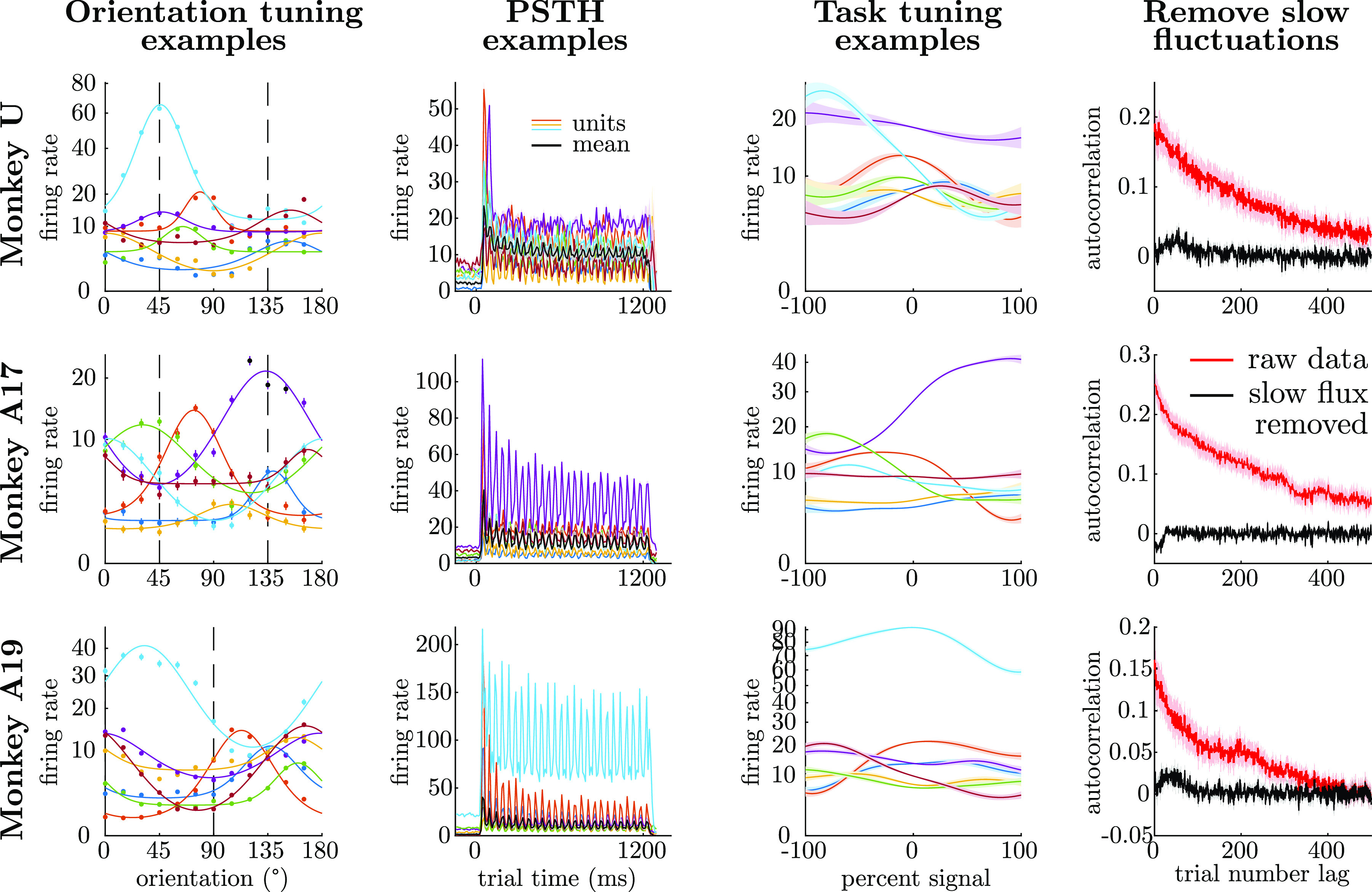
Example units from 1 session per experiment. Orientation tuning examples show firing rates for a subset of 7 randomly selected units in response to drifting gratings of varying orientations. Points show data ± SE; curves are von Mises tuning functions, fit by least squares to Anscombe-transformed spike counts after collapsing from 360° of grating drift directions down to 180° of orientations (few units were direction selective). The *y*-axis is scaled to be linear in the Anscombe-transformed rate but labeled by the equivalent firing rate. Vertical dashed lines show task orientations for the corresponding session (for comparison with task tuning examples). Summary of tuning for all units across all epochs can be found in Supplemental Fig. S5. Peristimulus time histogram (PSTH) examples show the PSTH for the same subset of units, binned at 10-ms resolution. Stimulus onset is at 0 ms, and offset is at 1,224 ms. Black line shows the mean response of all well-tuned units. Periodic fluctuations correspond to the 26 frames of the stimulus presented at 21.24 Hz. Task tuning examples show Gaussian process (GP) fits of the same subset of units’ responses to stimuli in the task, with 1σ uncertainty indicated by shading. GP tuning is also plotted linearly in units of Anscombe-transformed responses but labeled with equivalent firing rates. *Monkeys U* and *A17*’s data are taken from an Oblique session (−100% is 45°, +100% is 135°), and *A19*’s data are taken from a Cardinal session (−100% is 0°, +100% is 90°). Removal of slow fluctuation examples show the average autocorrelation of all well-tuned units across trials, before removal of slow fluctuations in red and after their removal in black (methods). Slow fluctuations were fit based on actual time rather than trial number but plotted here simply as a function of difference in trial number (i.e., not taking into account skipped trials or other long intertrial intervals).

Raw voltages were sampled and stored at 30 kHz, and spiking events were detected offline with custom methods designed to minimize confounds in estimating spike count correlations (methods). Spikes from all neurons on each electrode were grouped together as “multiunit” activity (referred to as “units” of neural activity throughout). A subset of sessions included drifting grating stimuli to assay tuning curves, and others included random block noise to assay receptive field locations (methods). Orientation tuning data were collected regularly for *monkey U* and *monkey A17* (Fig. 7, 1st column) but not for *monkey A19* because orientation tuning curves were assumed to have stabilized. However, we later found that our ability to predict neurons’ task sensitivity from their orientation tuning decayed over time, suggesting that analyses based on orientation tuning become less reliable. We therefore do not report any analyses for *A19* that require tuning curve properties such as units’ preferred orientations. Receptive field locations were stable throughout.

Example units are plotted in Fig. 7. Orientation tuning curves to drifting gratings, collapsed across drift directions to 180° of orientation, were typically well described by von Mises functions, plotted in the first column of Fig. 7. Peristimulus time histograms (PSTHs) showed stereotypical delays and onset transients, as well as additional fast transients in response to the 26 stimulus frames per trial, resulting in oscillating responses when averaged over many trials (Fig. 7, 2nd column). In the third column of Fig. 7, we show Gaussian process (GP) fits to each unit’s sustained response to different stimuli in the task (percent signal). Across the population, the average sensitivity of units in the task was well predicted by their orientation tuning curves (not shown), but the shapes of the tuning curves to percent signal in the task were often nonlinear or nonmonotonic. Tuning to percent signal was inferred jointly along with background slow fluctuations (methods). Estimated slow fluctuations with a timescale of 2 min were subtracted from neural responses, resulting in a dramatic reduction of autocorrelations across trials (Fig. 7, 4th column).

All models were fit to Anscombe-transformed spike counts to stabilize variance (60) (methods). Firing rates throughout Fig. 7 are plotted on the transformed scale but labeled with the equivalent mean firing rate.

### No Systematic Relationship between CP and Neural Sensitivity (d′)

Two important statistics linking neural activity to perception and decision-making are their neurometric sensitivity to the stimulus and their trial-by-trial correlation with the subject’s decision (23).

We measure the neurometric sensitivity of individual units (subscript *i*) as their signal-to-noise ratio at an arbitrary stimulus level of 50% signal, quantified using d′ (1):

$$d\prime_{i}\equiv 50f\prime_{i}/\sigma_{i}$$

*f′_i_* is an estimate of the derivative of the neural response to a unit change of percent signal in the task, estimated around zero signal. We estimated *f′* using only trials with signal strengths above 5.67%, we estimated noise covariance using trials below 5.67% signal, and we estimated CP using trials below 20% signal. We chose these ranges so that *1*) noise correlations and *f′* were estimated using disjoint trials to avoid confounds, *2*) noise correlations were estimated with as many low-signal trials as possible, and *3*) CP was estimated using as many trials as possible within a range where behavior was well described by classic signal detection theory methods and contained sufficiently many error trials (71).

We quantified individual units’ correlation with behavior using choice probability (CP), defined as the probability of predicting the subject’s decision based on whether the response of a unit exceeds a criterion (24, 26, 71). We report “signed” CP, which is 0.5 when there is no correlation between a unit’s response and the subject’s decision, above 0.5 when a higher response is correlated with “positive” choices, 90° or 135°, and below 0.5 when a higher response correlates with “negative” choices, 0° or 45°. Note that this differs slightly from convention; past work reports “unsigned” CP, which is equal to signed CP when d′ > 0 and 1 − CP when d′ < 0. This sign adjustment used in past literature discards critical information. By regressing “signed” CP against d′, not only are we able to test for the expected relationship but also additional patterns in CP and d′ may be identified; for instance, below we observe “one-sided” CPs independent of d′, which would be obscured in “unsigned” data. Significance of individual CPs was assessed by bootstrapping (resampling <20% signal trials with replacement). Two distinct theories predict, and numerous empirical studies have reported, a positive correlation between signed CP and d′ (as laid out in introduction). Note that the actual sign of both CP–1/2 and d′ depends on the arbitrary convention of assigning signs to orientations in each task, but the same convention is applied to both, so a positive relation between them is expected regardless.

We computed CP and d′ for each unit separately per session. We then combined data across sessions per epoch and tested for a relationship between CP and d′ using a regression model that explicitly accounted for two potential sources of statistical error: first, data for each regression analysis were collected across several days with chronically implanted electrodes, and so naively combining data across days would suffer from the problem of “pseudo-replication,” or treating multiple correlated measurements as independent. This would have the adverse effect of inflating the statistical significance of purely chance correlations and is addressed by performing linear mixed-effects (LME) regression. Second, standard LME regression does not account for measurement error, but here we have uncertainty in measurements of both CP and d′. To address both of these issues, we developed a LME regression model that jointly accounts for correlated measurements as well as uncertainty in both axes of the regression (methods).

**Figure 8.**
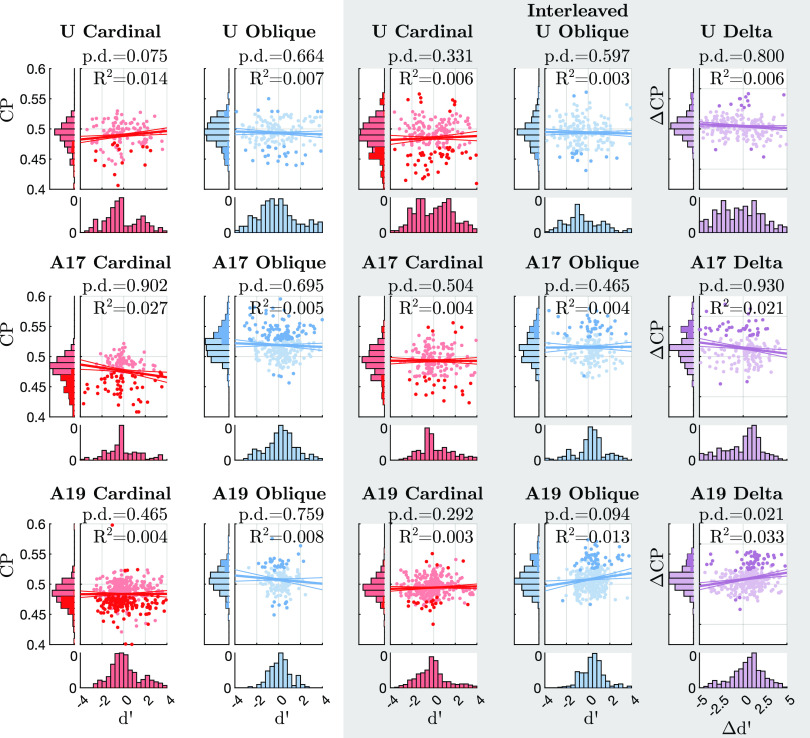
Little evidence for relationship between choice probability (CP) and neural sensitivity (d′) across epochs. We measured both CP and d′ of individual units, estimating uncertainty in each by bootstrapping (resampling trials with replacement, separately for trials used in each calculation). Regression was done with a hierarchical statistical model that accounted for structured variation in both CP and d′ across electrodes and days (methods). “p.d.” values are “probability of direction” (64), i.e., the posterior probability that the regression slope is negative. *R*^2^ values give the posterior median of marginal variance explained (methods; Refs. 62, 63, 65). *Columns 1–4* show scatterplots of CP vs. d′ in individual tasks, with marginal histograms of each. Darker shaded points and histograms correspond to individual CPs that are significantly on either side of 0.5 at *P* < 0.05 (bootstrapping). Overlaid lines indicate mean ± 68% credible interval of fixed-effect regression line. Inset text reports significance (fraction of posterior samples) of a 1-sided test that the slope is positive. In *column 5* (“Delta”), we regressed the change in CP between tasks vs. the change in d′. Histograms again depict the marginal distribution of ΔCP and Δd′. *Columns 3–5* (gray background) all come from interleaved sessions.

During initial task training and interleaved sessions, we tested for a relationship between CP and d′ separately for each task type (Fig. 8, first 4 columns). Furthermore, if CP depends directly on neural sensitivity in the task as predicted, then during interleaved sessions the change in neurons’ sensitivity from one task to the other should drive a change in CP. We therefore additionally tested for a relationship between the change in CP between tasks (ΔCP) and the change in neural sensitivity (Δd′) in data from interleaved sessions (Fig. 8, 5th column).

*Monkey U* has few units with individually significant CP, and only one of four conditions (initial Cardinal training) shows a weak but trending toward significant positive relation between CP and d′. Interestingly, in both of *monkey A*’s initial Cardinal and Oblique training epochs, we see a different pattern: nearly all significant CPs have the same sign, indicating that these units are collectively modulated up or down together depending on the monkey’s choice, regardless of the sign of their sensitivity to the stimulus, and this pattern of one-sided CPs continues into the third row for *A19*. Overall, we see no significant relationship between CP and d′ in any of *monkey A*’s data, with a slight exception in the comparison of ΔCP to Δd′ for *A19*, but it should be noted that none of these results is corrected for multiple comparisons. We therefore find no strong systematic evidence for a positive relationship between sensitivity and CP across our entire data set.

**Figure 9.**
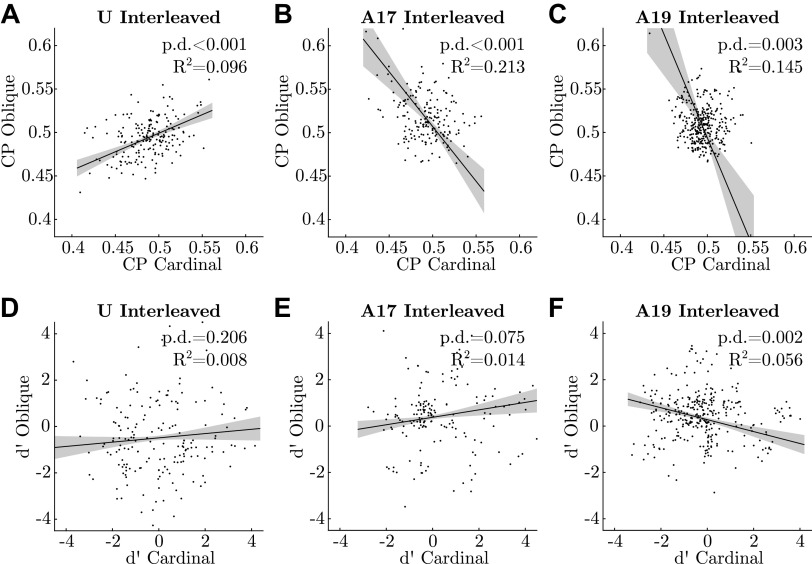
Comparison of choice probability (CP) and neural sensitivity (d′) across tasks in interleaved sessions using the same hierarchical regression model as in Fig. 8. accounting for correlations across sessions and across electrodes. Regression lines show mean ± 68% credible intervals from the posterior over fixed-effects regression parameters (methods). The 2-sided probability of direction (“p.d.”; Ref. 64) and posterior median value of marginal *R*^2^ (methods; Refs. 62, 63, 65) are shown. See Supplemental Figs. S8 and S9 for plots split by random effects (individual sessions and electrodes). *A–C*: significant correlations between signed CP values across tasks. Note that the sign of the regression only reflects our arbitrary analysis choice of associating 0° and 45° with the “negative” direction. Note that results for *monkeys A17* in *B* and *A19* in *C* may be driven to a large degree by outliers with low Cardinal and high Oblique CP. *D* and *E*: units are independently sensitive (d′) to the Cardinal or Oblique task in *monkey U*’s and *monkey A17*’s data, indicating that we have an unbiased sample of units. *F*: slight correlation between d′ in each task in *monkey A19*’s data. This is likely due to chance placement of the electrodes and which units survived our exclusion criteria during preprocessing; this relationship was not robust to changes to our preprocessing methods (see methods).

Another possibility is that choice information lives in the same subspace in both tasks rather than aligning with d′ separately per task (46). To test this, we regressed CP in the Oblique task against CP in the Cardinal task during interleaved sessions. We used the same regression model as before, accounting for both uncertainty in measurements on both axes as well as correlated measurements from chronic electrodes across days. This analysis indeed revealed a significant correlation between CPs across tasks (Fig. 9, *A*–*C*). We additionally tested for correlations in d′ across tasks and found none in *monkey U*’s or *monkey A17*’s data (Fig. 9, *D* and *E*), but a slight relationship in *A19*’s data (Fig. 9*F*). Taken together, these results appear consistent with the idea that the “choice axis” depends on task-independent factors and is not strongly affected by neural sensitivity on each task, at least not on short timescales (46).

Overall, we find few strong individually significant CPs. We also considered the possibility that choice-related information is distributed across the population but not strong in individual units. We used a regularized variant of linear discriminant analysis (LDA) to find the optimal “choice decoder” for each session. We then tested whether we could decode the monkeys’ choices from the projection of population activity onto the decoder axis in held-out trials (methods). The use of regularization and held-out trials in LDA is crucial: it allows us to estimate the amount of choice information in the population robustly across trials and in an unbiased way (72). We found these population choice decoders to be in agreement with the conclusions from single-unit CP analyses (Supplemental Fig. S10): decoding *monkey U*’s choice from the population was not significantly above chance in most sessions, an exception being during Cardinal trials of the interleaved phase. Again consistent with the single-unit CP analysis, *monkey A* showed small but significant choice information in the population commensurate with the size of individual CPs in most epochs. However, we again see little significant choice information remaining in *A19*’s data during the Interleaved epoch. See Supplemental Fig. S11 for neurometric decoder performance using the same method, which revealed robust stimulus information in all sessions.

### Analysis of Noise Correlations

**Figure 10.**
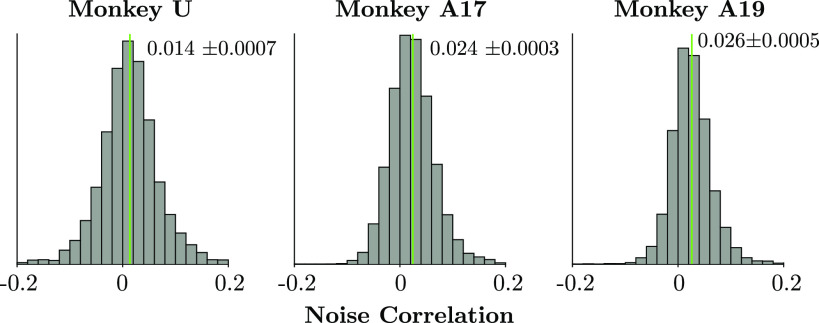
Noise correlations histograms for each monkey. We collapsed across sessions and epochs because no significant differences were found between them. Text and green lines indicate mean correlation ± SE.

We next turned to the question of whether task training induced structure in the pairwise noise covariance between units, as has been previously found (41, 47). Noise covariance was computed by subtracting the mean Anscombe-transformed response to get “residual” responses separately per signal level and pooling these residuals up to ±5.67% signal before computing their empirical covariance. Additionally conditioning means on specific noise seeds (i.e., restricting the analysis to the very same “frozen” noise stimulus) did not significantly impact our analyses. For comparison with prior results, we first converted covariances to correlations and report their distribution (Fig. 10). Average noise correlations for *monkey U* were 0.014, for *monkey A17* they were 0.024, and for *monkey A19* they were 0.026. Previous studies vary in their estimates of noise correlations in macaque V1 (9), but most that report larger correlations do so in anesthetized animals, where slow background fluctuations are especially high (18). It has also been shown that shared fluctuations in local field potential (LFP) can inflate estimates of spike count correlations in extracellular electrodes (56), which we mitigate by whitening raw voltage signals before detecting spikes (methods). Our small mean correlation estimates are in line with those in V1 of awake monkeys reported by Ecker et al. (73), who analyzed well-isolated single units.

**Figure 11.**
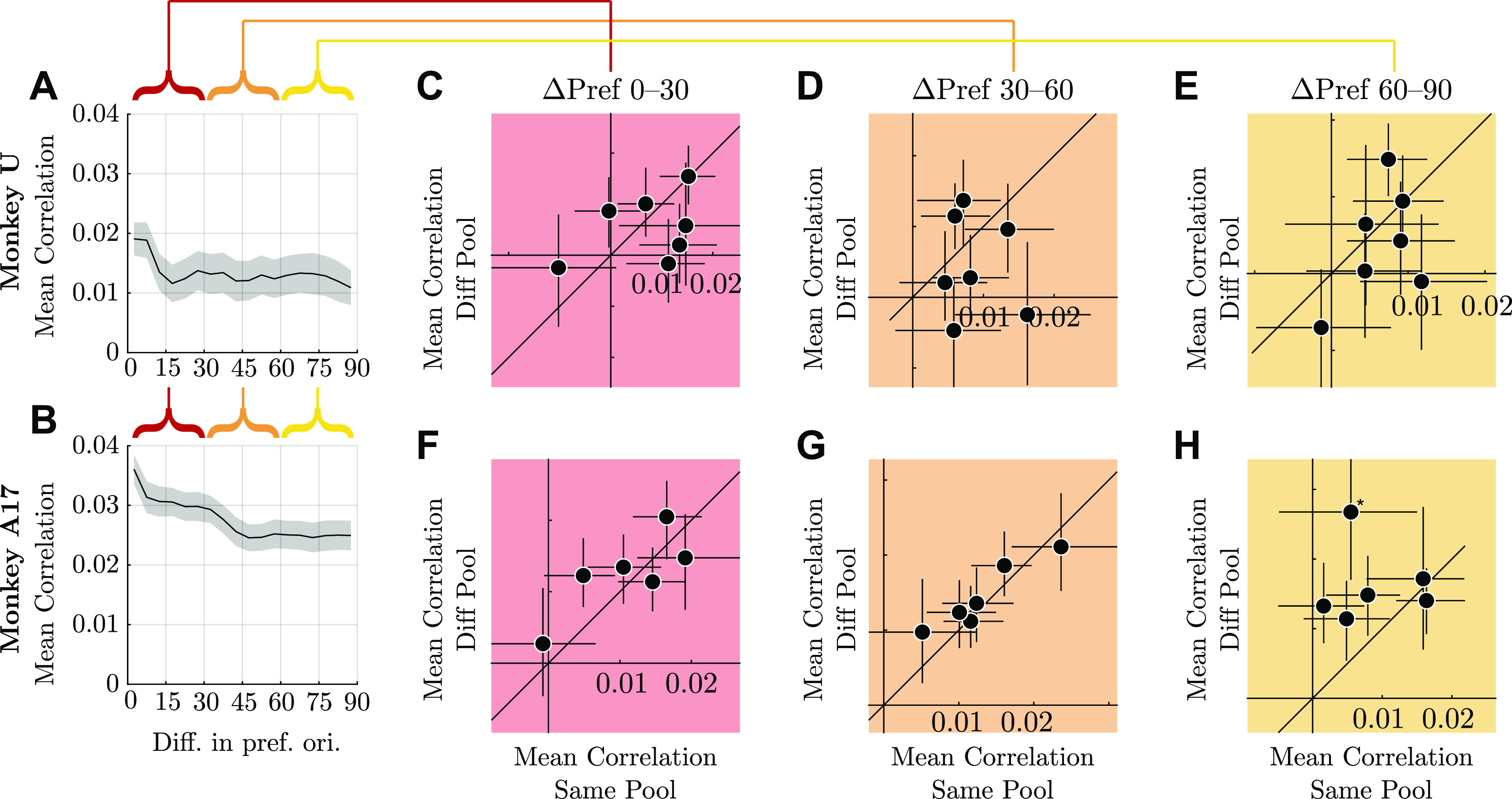
Limited range correlation structure shows no dependence on decision pool, failing to generalize (47) to V1 neurons for an orientation-discrimination task. *A* and *B*: noise correlations combined across all epochs and sessions, plotted as a function of the difference in preferred orientation of each pair of units. Shaded areas indicate SE with data binned at 5° increments. *Monkey U*’s noise correlations exhibit a slight drop after a difference of 10° but remain flat afterward. *Monkey A*’s noise correlations exhibit a downward trend out to a difference of 45^∘^. *C–H*: each point corresponds to a single session, and error bars indicate SE. The “pool” for unit *i* is determined by the sign of neural sensitivity (d′*_i_*), and here we analyze only the subpopulation of units whose pool switched between tasks. We tested for a difference between average “same-pool” correlations and average “different-pool” correlations, separately for each of 3 bins: pairs with small (red), medium (orange), or large (yellow) differences in preferred orientation. We found no statistically significant difference in mean correlations between pools in all but a single session at the largest difference in preferred orientations (*H*).

Neurons with more similar tuning preferences are expected to be more correlated with each other, in part because they share afferent noise (8, 47). One way this is classically quantified is with so-called limited range (LR) correlation structure: noise correlations are expected to decay as a function of the difference in the pair’s preferred orientations, measured from orientation tuning sessions. Indeed, we find such a downward trend in both *monkeys U* and *A17* (Fig. 11, *A* and *B*). Data from *A19* are excluded from this analysis because we lack reliable estimates of units’ preferred orientations.

Cohen and Newsome (47) were the first to report task-dependent changes in noise correlation structure during cued interleaved discrimination tasks (in area MT). When two neurons’ tuning to the task have the same sign (i.e., both d′ > 0 or both d′ < 0), they are considered part of the same “pool” for the decision, whereas neurons with opposite-sign tuning are considered to be in different pools (27). By cuing the monkey to the task at the start of each trial, Cohen and Newsome (47) tested whether the same pair of neurons’ correlation was modulated in virtue of being part of the same or different pool and found a clear division in LR correlation structure between the two. In particular, they found stronger correlations within the “same pool” units at small differences in preferred orientation, but the difference reversed for larger differences in preferred orientation (see Figs. 3 and 4 in Ref. 47).

We tested for this trend during Interleaved epochs for *monkeys U* and *A17* by computing the mean correlation within each pool, separately for three bins of differences in preferred orientations (*A19* data are again excluded). In our data, we found no systematic differences in correlations between decision pools at any range of differences in preferred orientation (Fig. 11, *C*–*H*). We therefore find no evidence that the decision pool-related correlation structure discovered by Cohen and Newsome (47) for MT neurons (using a direction-of-motion discrimination task) generalizes to V1 neurons for animals performing an orientation-discrimination task. In this analysis, we tested for significance by bootstrapping trials and computing the fraction of paired differences (same pool in one task and different pool in the other) that were on either side of zero. One session from *monkey A17* reached significance, with different-pool correlations larger than same-pool, only in the bin of large differences between preferred orientations (Fig. 11*H*).

### No Task Dependence in Noise Covariance

**Figure 12.**
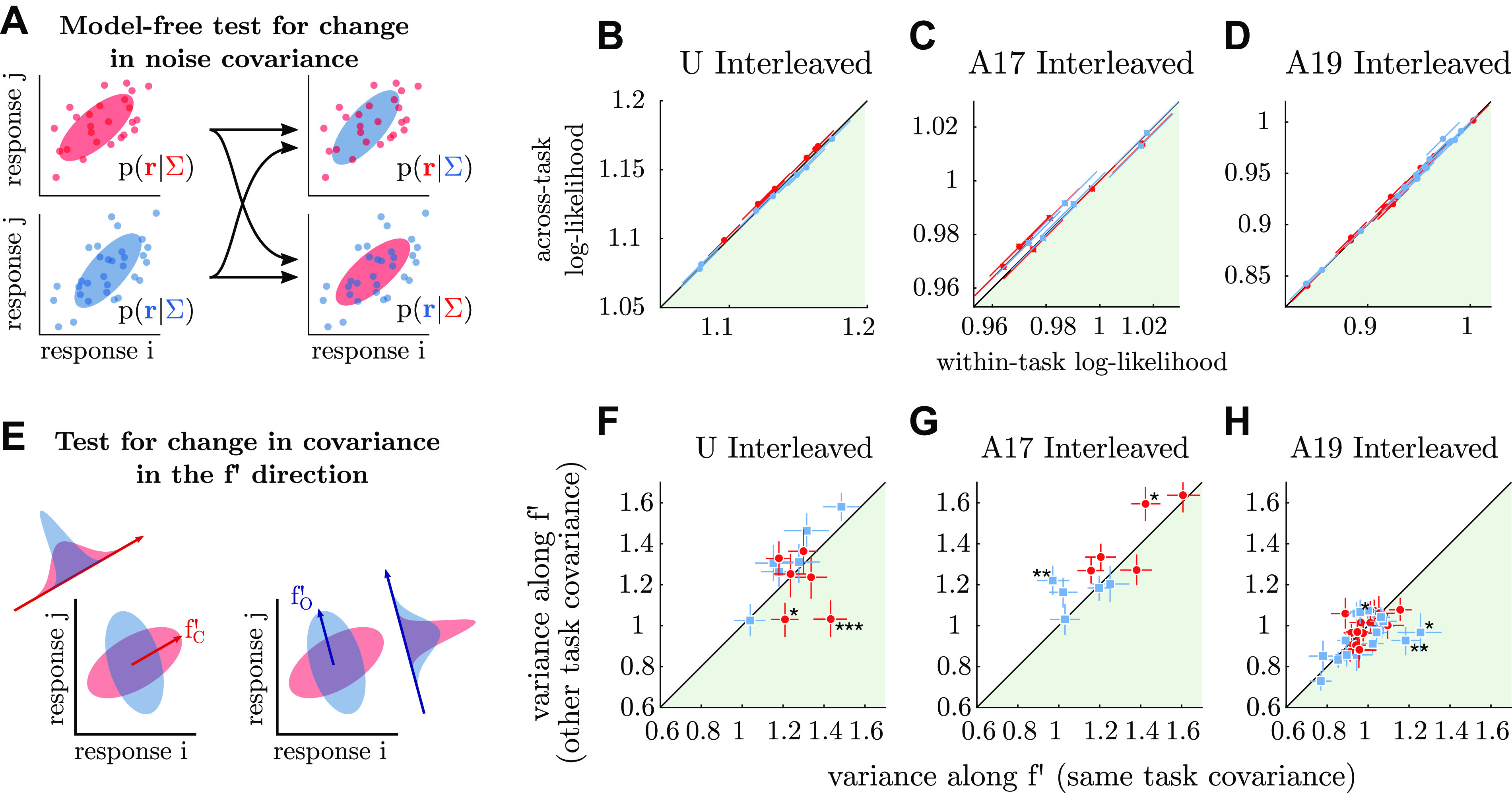
Two tests for change in noise covariance between tasks. *A*: we first used a model-free method to test for change in noise covariance between tasks: comparing the per-trial predictability (log likelihood) of held-out spikes in each task using either a covariance matrix fit to separate training trials from the same task or a covariance matrix transferred from training trials in the other task. All training and test trials are <5.67% signal. *B–D*: scatters of across-task prediction scores vs. within-task prediction scores, 2 data points per session [1 Cardinal (red) and 1 Oblique (blue)]. Error bars indicate SE across train/test splits, computed along the *y* = *x* and *y* = −*x* axes to highlight the difference in log likelihoods. If there were significant changes in covariance structure between tasks, we would expect to see higher within-task prediction than across-task (green shaded region) performance, but no sessions reached statistical significance in this direction. *E*: to test the specific hypothesis that noise covariance may change by adding a component in the **f′** direction for each task, we projected population responses onto the Cardinal **f′** and Oblique **f′** directions in neural space and then tested for a change in the variance of each projection between tasks during interleaved sessions. For each task’s **f′** direction, we computed the difference between the projected covariance from the same task and that from the other task. If noise covariance aligns with **f′** separately for each task, then the “same” projection should be larger than the “other” projection, as illustrated here and in the gray shading in *F–H*. *F–H*: each session gives 2 points: 1 for the Cardinal and 1 for the Oblique task. Asterisks indicate individually significant sessions, and error bars indicate 68% confidence intervals, estimated by bootstrapping both **f′** and the noise covariance matrix. Excepting single tasks for a few outlying sessions, we find no systematic evidence that noise covariance increases along **f′** for each task.

To test for changes in noise covariance structure between tasks, we first developed a model-free measure of the amount of change in covariance between two conditions. If there is any substantial change in noise covariance between tasks during interleaved conditions, then the responses of neurons in held-out trials from one task should be better predicted by a model fit to trials from the same task context than they are by a model fit to trials from the other task (Fig. 12*A*). We quantified this difference using the average log-likelihood of held-out trials under a Gaussian model, using a regularized estimate of the covariance matrix (methods). Across all data splits, all interleaved sessions, and in all three experiments, no sessions showed significantly higher predictability within versus. across tasks (Fig. 12, *B*–*D*). This analysis rules out large systematic changes in covariance between tasks. However, such changes may nonetheless be present but undetectable with this method. This analysis is “model free” because it does not assume any functional form of possible changes, such as changes that align with the $f\prime f^{\prime ⊤}-$ direction for each task (37, 38). The added flexibility of a model-free test comes at the expense of statistical power. We therefore turn next to an explicit test for changes in noise covariance along $f\prime f^{\prime ⊤}$ directions.

We further tested for changes in variability along the **f′−** direction specifically by computing the difference in the amount of variance along each task’s **f′** axis using neural responses from each task (Fig. 12*E*). If $f\prime f^{\prime ⊤}$ covariance is present and large during the concurrent task, then the amount of variance along the **f′** direction corresponding to the Cardinal task should be reduced during the Oblique task, and vice versa. We computed differences in variance along **f′** separately per task and per session (Fig. 12, *F*–*H*). Consistent with the previous model-free analysis, we see a few statistically significant points for individual tasks and individual sessions, but none indicates significant changes aligned with **f′** for both tasks on the same day. Aggregated across sessions, then, we find no statistically significant differences between within- and across-task variance along **f′**.

## DISCUSSION

### Summary

Understanding the nature of decision-related signals in sensory areas is crucial for understanding sensory information processing. Motivated by converging lines of evidence from both theoretical and empirical work that linked choice probabilities (CP) and noise correlations to feedback signals, we set out to replicate and extend previous results on their task dependence. We attempted to track neural correlates of task-switching in primary visual cortex of two monkeys trained on a pair of orientation-discrimination tasks. Not only did we fail to find robust neural signals related to task-switching, but we also found only weak evidence for the kinds of choice-related signals that have been widely reported in the literature for similar perceptual learning tasks. In the following, we consider various possible explanations for this somewhat puzzling lack of evidence. These include experimental issues, such as task training, neural populations, and stimulus design that differed between our and prior studies, as well as differences in data preprocessing and various analysis choices. Finally, we attempt to make sense of our results in the context of previous results. We believe these comparisons provide insights into the robustness and ubiquity of choice-related signals and task-dependent noise correlations and constrain future models of how they arise.

### Was Our Experiment Appropriate for Testing Our Hypothesis?

In this section, we consider critical experimental factors that are necessary for the data to be useful in addressing our hypothesis that trial-by-trial task-switching induces changes to noise correlations and choice probabilities in V1. We review the evidence that our animals indeed switched tasks (for at least 2 of the 3 data sets) and that our neuronal sample and visual stimuli were appropriate, although we also raise some caveats and considerations for future work.

#### Did our monkeys switch tasks?

In two of three data sets collected with randomly interleaved task types, *U* and *A19*, we found strong evidence that the animals used different strategies for the two different tasks (Fig. 5). Furthermore, whatever strategy they used, the monkeys’ performance was stable (Fig. 3; Supplemental Fig. S1). We thus think it unlikely that a lack of task learning can account for our negative neural results.

#### Did we record from the right neurons?

Two lines of evidence suggest that our neural sample was not the reason for a lack of positive results. First, unlike other studies using chronic multielectrode arrays (MEAs), we were able to obtain postmortem histological assignment of the laminar locations of most of our electrodes (Fig. 6; Supplemental Fig. S4). We were somewhat surprised to find in *monkey U* that some of our electrodes were deeper than we would have thought possible, with approximately one-third of them in layer 4B or below (Fig. 6*C*). Nevertheless, we are confident that the majority of our recordings in *monkey U* and all of them in *monkey A* were from supragranular layers, which provide the main source of feedforward outputs to higher cortical visual areas. It remains an interesting possibility that choice-related signals are stronger in infragranular cortex, but we know of no other comparable studies in the monkey that have reported laminar information.

The second line of evidence is that the properties of the neurons from which we recorded were well suited to provide task-relevant signals. They were well tuned for orientation (Fig. 7; Supplemental Fig. S5) and showed task-related tuning that was on par with those reported in previous studies (Supplemental Fig. S6). Furthermore, the pooled sensitivity of our recorded neural population was sufficient to account for the psychophysical sensitivity of our monkeys (Supplemental Fig. S7) (23).

It remains a possibility that primary visual cortex (V1) was not the right visual area from which to record signals related to task-switching. Two previous studies also largely failed to find choice-related signals in macaque V1 during orientation-discrimination tasks (44, 45). However, both of these studies used a fine-discrimination task, in which animals judged small orientation differences of high-contrast gratings relative to a fixed discrimination boundary. Although there are other differences between these studies and ours, the fine versus coarse nature of the task may be a critical one; low-signal stimuli in a coarse-discrimination task are “open to interpretation” in a way that may make them more susceptible to feedback-induced biases (38, 74). Moreover, the experiments of Bondy et al. (41) and Nienborg and Cumming (42), which were similar to ours in being a coarse-discrimination task with noise and which used the same class of visual stimuli (but see below), did find choice-related signals and changes in correlation structure that followed task contingencies in V1 neurons. We thus believe that recording from V1 was not the likely cause of our negative findings.

#### Stimulus match to neuron preferences.

Did subtle differences in the relationship between our visual stimuli and neural receptive fields account for differences with previous studies? Although our visual stimuli were nominally identical to those used by Bondy et al. (41), taken directly from the psychophysical study of Beaudot and Mullen (69), subtle differences in relative stimulus size may have played a role. Because we sought to cover the aggregate receptive fields on the MEAs, our stimuli were relatively larger than those used by Bondy et al., and thus may have more strongly activated the suppressive surrounds known to be a feature of the receptive fields of most V1 neurons (75–77). This difference could account for some of the nonmonotonic task-tuning curves we saw (Fig. 7) insofar as the dynamic noise stimulus differentially engaged center versus surround mechanisms (78). More importantly, it could have contributed to the relative lack of choice probability we observed: Kang and Maunsell (79) showed that, for a visual detection task, choice-related correlations grew stronger as the stimulus size and spatial frequency better matched the receptive field properties of a given area.

Interestingly, this possibility is consistent with between-monkey differences in Bondy et al. (41). They used two different recording technologies for the two monkeys in their study: in one animal (“JBE”) they used the same (Utah) arrays that we used in both of our monkeys, meaning that receptive fields were relatively spread out and more likely to be different in preferred orientation and other dimensions; in the other monkey (“LEM”) they used laminar V probes that recorded neurons within a single column, thus sampling receptive fields that were much more similar and for which the stimulus could therefore be better tailored. It was in the second, V-probe monkey that they found stronger choice-related signals, consistent with the results of Kang and Maunsell (79). Note, however, that monkey LEM had also been trained for much longer (see below) and showed better overall performance on the task, preventing a firm conclusion as to whether task-training, better tailoring of the stimulus to receptive field (RF) properties, or both accounted for the difference between their monkeys.

### Methodological Considerations

We next consider several analytical issues that are important for comparing our work with previous studies.

#### Neural data preprocessing.

Numerous previous studies have reported noise correlations in various brain areas using Utah arrays. It is known that fluctuations in local field potential can trigger false-positive spiking events and that noise correlations are inflated by misattributing spikes across electrodes (9, 56, 73). To our knowledge, no previous study using Utah arrays included whitening voltage signals across channels as a preprocessing step as we do here [methods; inspired by analogous preprocessing in Kilosort (55)]. In earlier analyses, we found that, in the absence of whitening, false positives in spike detection significantly inflated correlation estimates broadly, and even resulted in spurious $f\prime f^{\prime ⊤}$-like structure during Cardinal trials in *monkey U*. Although we have removed low-level spiking confounds to the best of our ability, these observations point to the need for robust and standardized data processing tools for extracellular array data (80).

#### Repeated measures and hierarchical regression.

Repeated experiments across multiple days with chronic arrays increase statistical power, but correlations among data points complicate statistical tests. Although most off-the-shelf statistical tools assume independence of all data points, there are day-to-day correlations in the measurements from array data that need to be taken into account (81): not only are measurements from the same electrode correlated across days, but measurements on each day are correlated across electrodes. Treating longitudinal array data as independent risks pseudo-replication, which spuriously inflates estimates of statistical significance. We used a linear mixed-effects (LME) model to address this for analysis of CP and d′ data (methods) and found that multiple puzzling and seemingly significant initial results vanished. Still, with this method we find some significant correlations that do not generalize across learning epochs or monkeys (e.g., between the CPs for different tasks; Fig. 9). This heterogeneity of results suggests that we may underestimate our uncertainty over quantities of interest even when using hierarchical regression methods applied to lower sensory areas. Accounting for pseudo-replication in our later analyses of noise covariance is even more challenging (naively requiring a separate “random effect” parameter for each unique pair of units, which we do not have enough data to estimate). This is why we instead report all analyses of noise covariance separately for individual sessions.

#### Statistical power to detect changes in noise covariance.

We tested for task-dependent changes in noise covariance or noise correlation structure three different ways: by testing for changes in limited-range correlations as done by Cohen and Newsome (47), by testing for “model-free” changes in covariance, and by testing for task-specific changes in the **f′** direction; all tests yielded negative results. It is possible that we simply lacked statistical power to detect small effects that are, in fact, present. Rumyantsev et al. (15) showed that one’s ability to recover low-rank factors from a population of neurons depends on the number of trials, the number of neurons, the number of latent factors, and the variance of those latent factors. Crucially, it was shown that as the amount of data is reduced, one’s ability to detect a latent factor does not degrade smoothly but undergoes a rapid phase transition from partially detectable to completely undetectable. This confounds our ability to track these changes over the course of learning. Future studies using denser recording methods, such as Neuropixel probes, should help to resolve this issue.

### Making Sense of Largely Negative Results

On the whole, we found only weak evidence for choice-related signals in V1 and no evidence for neural correlates of task-switching. In this section, we speculate on possible reasons, some of which suggest a different interpretation of choice-related neural activity in early sensory areas.

#### Unexpected alignment of stimulus and choice axes.

We found a small but significant relationship between CP and d′ in the Cardinal task data for *monkey U*, but this relationship disappeared for the Oblique task (Fig. 8). In a subsequent analysis, we found that for the same monkey CP but not d′ was highly correlated between the two tasks (Fig. 9, *A* and *D*), suggesting that choice information lay primarily along a single axis in both tasks (46). This may explain why we found a significant correlation between CP and d′ in only one task: although choice information lies along the same axis in both tasks, this “choice axis” is partially aligned with neural sensitivity in the Cardinal task but orthogonal to the direction of sensitivity in the Oblique task. Given behavioral evidence that *monkey U* switched his strategy between tasks, it is unclear why choice information would align with **f′** for only one task. One possibility, in line with the discussion on perceptual learning (see below), is that this reflects the monkey’s initial training in the Cardinal task (Fig. 3).

*Monkey A*’s CPs showed different structure: nearly all (signed) CPs differed from 0.5 in the same direction. This may indicate that choice information for *monkey A* correlates with the overall activity level of a subpopulation of units, an observation consistent with the types of biased representations seen in multiple different studies in posterior parietal cortex (82). This is partially replicated in *monkey A*, where we see a significant (negative) correlation between CP across tasks (Fig. 9, *B* and *C*), and among units that changed CP between tasks significant changes were all one-sided (seen in the marginal histogram of ΔCP in Fig. 8). However, this pattern of results is weaker in *monkey A* than in *monkey U* and may be driven by a relatively small number of outlying units. Taken together, the one-sided CPs in *monkey A*’s data, along with *monkey U*’s results, suggest that choice information may lie along a single task-independent axis for both monkeys (46).

#### Results with alternative analysis methods.

Although our data analysis methods are designed to minimize confounds while maximizing our potential to find effects that are truly present, they are nonstandard. To address this, we changed how neural responses were computed and reran all of our main analyses after the first version of this article was written. Whereas in our main results neural responses are defined as Anscombe-transformed spike counts with slow fluctuations subtracted, in this new analysis neural responses are defined as the average spike rate after stimulus onset, with the baseline spike rate in the prestimulus interval subtracted on a trial-by-trial basis. Subtracting the baseline activity on a trial-by-trial basis in this way is standard but often unspoken practice. Ultimately, these “baseline-subtracted” data further supported our main conclusions. In particular, we found *1*) no significant trend in the CP vs. d′ relationship as in Fig. 8, *2*) slightly stronger evidence for a relationship between CP in the Cardinal task and CP in the Oblique task as in Fig. 9, *A*–*C*, *3*) no significant relationship between d′ in the Cardinal task and d′ in the Oblique task, as in Fig. 9, *D*–*F*, and *4*) no evidence for task-specific changes to noise covariance, as in Figs. 11 and 12.

#### Constraint on timescale of task-specific feedback.

As noted above, our experiment is most closely related to that of Bondy et al., who reported significant task-dependent changes in noise correlations across coarse orientation discrimination tasks with stimuli similar to ours and further found them to be aligned with $f\prime f^{\prime ⊤}$. The most salient difference between the two studies is that whereas we trained monkeys to switch between tasks on a trial-by-trial basis, Bondy et al.’s monkeys performed only a single task per session, alternating between them by relearning each task over the course of several days. It has been previously argued, both by the original authors and by us (38), that retraining over the course of a few days is unlikely to change feedforward or lateral connectivity in V1, suggesting that their reported changes in noise correlation structure are indeed due to feedback (41). It may be the case, however, that task-specific feedback cannot be rapidly switched from one trial to the next, at least not feedback signals to area V1, in our task (however, see Ref. 47 for a counterexample based on area MT and a direction discrimination task).

#### Task learning vs. perceptual learning.

A second difference between our experimental design and that of Bondy et al. (41) is that our animals were not as extensively trained as theirs were at the time that neural recordings were made. Because we had hoped to also track choice-related signals as animals learned the tasks, we changed task versions soon after our monkeys showed stable performance (Fig. 3) and then began interleaving the two tasks in blocks of diminishing sizes immediately after that. Bondy et al. (41), on the other hand, trained their animals extensively before neural recordings began: one of their animals (LEM) performed the same task in an earlier study (42) and thus had >2 yr of prior training; the second monkey (JBE) had >1 yr of experience with the task (A. Bondy, personal communication). Furthermore, LEM showed substantially larger effect sizes than JBE. The study of Cohen and Newsome (47) was similar in this regard: one monkey was a “laboratory veteran” who had performed the psychophysical task for a previous project, and the other had been trained for 6–9 mo before electrophysiological recordings (M. Cohen, personal communication). It remains a possibility that the influence of idiosyncrasies in each subject’s training history on the relationship between sensory and decision-making areas complicates comparisons across studies (83, 84). Furthermore, in the one study of which we are aware that reported changes in choice-related signals during learning (39), the relationship between neurometric sensitivity in an early sensory area (MT) and choice probability evolved over 120 sessions and was very weak, barely reaching statistical significance in the final one-third of the sessions. This raises the interesting possibility that choice-related signals in early sensory areas are less indicative of task learning and instead are more closely related to perceptual learning that occurs in overtrained subjects.

### Concluding Remarks and Outlook

We set out to replicate and extend prior findings on choice probabilities and noise correlations. Unfortunately, we failed to replicate key prior findings on the relationship between CP and neural sensitivity that are believed to hold widely across neurons involved in a perceptual decision-making task. Our study was not an exact replication of any of the previous studies and therefore does not cast doubt on these prior empirical findings. Instead, it adds to the growing body of work that has failed to find robust choice probabilities or their relationship with neural sensitivity. Our results further place constraints on how well prior findings generalize across tasks, stimulus parameters, training stage, or different groups of neurons being sampled by different recording technology. Since many of these aspects are highly correlated across existing studies, future work will need to systematically explore each aspect’s effect on choice probabilities and noise correlations, in turn placing tighter constraints on theoretical models.

## DATA AVAILABILITY

All (preprocessed) data required to reproduce the results in this paper are available publicly online at https://osf.io/nfta6/. Additional (raw) data will be made available upon reasonable request.

### SUPPLEMENTAL MATERIALS

Data, code, and supplemental text, figures, and tables are available publicly online at https://osf.io/nfta6/.

## GRANTS

This work was funded by NIH R01 Grant EY028811 to R.M.H. and R.T.B., as well as National Science Foundation (NSF) Traineeship in Data Science 1449828 and NIH Traineeship in Vision Science 5T32EY007125 to R.D.L.

## DISCLOSURES

No conflicts of interest, financial or otherwise, are declared by the authors.

## AUTHOR CONTRIBUTIONS

C.G.-L., R.M.H., and R.T.B. conceived and designed research; C.G.-L., V.K.B., and A.S. performed experiments; R.D.L., C.G.-L., and T.H. analyzed data; R.D.L., A.P., R.M.H., and R.T.B. interpreted results of experiments; R.D.L., V.K.B., T.H., and R.T.B. prepared figures; R.D.L., R.M.H., and R.T.B. drafted manuscript; R.D.L., A.P., R.M.H., and R.T.B. edited and revised manuscript; R.D.L., C.G.-L., V.K.B., A.P., A.S., T.H., R.M.H., and R.T.B. approved final version of manuscript.
